# Supplementary material for: Comparing the reliability of relative bird abundance indices from standardized surveys and community science data at finer resolutions
Source: PLoS One. 2021 Sep 10;16(9):e0257226. doi: 10.1371/journal.pone.0257226 (PMC8432801; doi:10.1371/journal.pone.0257226)
Supplement: S1 Appendix — Details on the random forest method used to estimate the breeding season detection probability of 14 bird species in Massachusetts. (ZIP) [file pone.0257226.s001.zip › S1_Appendix.pdf]

# S1 Appendix. Random Forest Detection Probability Methodology

The Best Practices for Using eBird Data [1], outlines how to reliably estimate species distributions using the R statistical language and data from the eBird Basic Dataset [2]. We reference their methodology for estimating species ”encounter rates” which they define as the probability of encountering a species on a standardized eBird checklist. We interpret these encounter rates as detection probabilities and adapt their modeling framework to align with the spatial and temporal scale of our available data. All data preparation and analysis were performed in R 4.0.2 [3].

To calculate breeding season detection probability, we used the filtered and sub-sampled Massachusetts eBird detection data described in our Materials and methods section. The sub-sampling, referenced from the eBird Best Practices, reduced spatial and temporal bias from eBird's opportunistic sampling as well as class imbalance due to the large proportion of non-detections [1]. We used the R-package *dggridR* [4], to overlay a grid of equal area hexagonal cells with 5km spacing on the study area of Massachusetts. We randomly sampled one detection and one non-detection from each grid cell for each week of the time period. This process increased the detection prevalence which increased the detection probability of the model predictions. We balanced sample sizes across years by calculating the average number of checklists in each breeding season and re-sampling checklists in each year to meet this average number [5]. In later years with higher than average checklist submissions, checklists were randomly re-sampled without replacement. Earlier years with lower than average checklist submissions were randomly re-sampled with replacement. This sub-sampling was applied to the eBird data used for both the GLMM and RF methods.

The data was then split into testing (20%) and training data (80%), before running a random forests (RF) model with the R-package *ranger* [6]. The variables used in the model were species detection as a response to temporal variables (year, day of year, and starting time of observations) and effort variables. The multiple temporal variables account for year to year variation in abundance, changes in species abundance and detectability throughout the breeding season, and changes in species and observer activity throughout the day. We started with multiple effort variables as suggested by the eBird Best Practices, but also included list length (the number of species detected on a checklist survey) as an additional effort covariate. This is because it was shown to be representative of multiple effort variables in previous eBird modelling applications [7, 8]. We verified this finding using the RFs ability to assess variable importance through ranking variables by average Gini coefficients [1]. List length was the most important effort variable consistent across all species. Therefore, we only use list length as an effort variable in the final model (removing survey duration, distance, and number of observers), as we found little change in model performance compared to including all effort variables. Town ID was included in the model to account for variation in habitat availability.

We used a balanced RF approach by including the *sample.fraction* parameter in *ranger* because class imbalance towards non-detections still remained in the data. This approach samples an equal proportion of detections and non-detections in each random sample. A calibration of the predictions was used to diagnose and realign the predicted encounter rates with observed detection probabilities. The calibrations were calculated with generalized additive models (GAM) fit with the observed probabilities as the response to predicted probabilities and constrained only to increase since we a priori expect predictions to increase with observations. We assessed model accurracy using the testing data and predictive performance metrics (mean squared error, sensitivity, specificity, AUC, and kappa). We maximized the threshold for the classification of

probabilities into binary detections/non-detections, required in the model assessment [1].

Town-level breeding season detection probabilities and their standard errors were predicted for each species by using aannual, town prediction dataset. We standardized the prediction effort variables so they represented one observer conducting an hour long survey, traveling 1km at the peak time of day and the peak day of each month. The peak time of day for each species was calculated from the partial dependence of time of day on detection. The peak day of each year was calculated as the day within the breeding season with maximum detection frequency across the whole training dataset. List length was used as the mean list length across the detection data. State-level estimates were calculated as the weighted average of town-level estimates in each year. Town estimates were weighted by the inverse of their SEs to place less weight on towns with greater uncertainty.

## References


1. Strimas-Mackey M, Hochachka WM, Ruiz-Gutierrez V, Robinson OJ, Miller ET, Auer T, et al. Best Practices for Using eBird Data. Version 1.0. Cornell Lab of Ornithology, Ithaca, New York; 2020. Available from: `https://cornelllabofornithology.github.io/ebird-best-practices/`.
2. eBird Basic Dataset. Version: EBD_relMay-2020; 2020.
3. Team RC. R: A Language and Environment for Statistical Computing; 2020. Available from: `http://www.R-project.org/`.
4. Barnes R. dggridR: Discrete Global Grids. R package version 2.0.4.; 2020. Available from: `https://CRAN.R-project.org/package=dggridR`.
5. Fink D, Auer T, Johnston A, Ruiz-Gutierrez V, Hochachka WM, Kelling S. Modeling avian full annual cycle distribution and population trends with citizen science data. Ecological Applications. 2020;30(3):e02056. doi:10.1002/eap.2056.
6. Wright MN, Zeigler A. ranger: A Fast Implementation of Random Forests for High Dimensional Data in C++ and R. Journal of Statistical Software. 2017;77(1):1–17. doi:doi:10.18637/jss.v077.i01.
7. Horns JJ, Adler FR, Sekercioglu CH. Using opportunistic citizen science data to estimate avian population trends. Biological Conservation. 2018;221:151–159. doi:10.1016/j.biocon.2018.02.027.
8. Walker J, Taylor P. Using eBird data to model population change of migratory bird species. Avian Conservation and Ecology. 2017;12(1). doi:10.5751/ACE-00960-120104.
