# Supplementary material for: Comparing the reliability of relative bird abundance indices from standardized surveys and community science data at finer resolutions
Source: PLoS One. 2021 Sep 10;16(9):e0257226. doi: 10.1371/journal.pone.0257226 (PMC8432801; doi:10.1371/journal.pone.0257226)

S2 Appendix. **Correlation matrices of four relative abundance indices within and between datasets.** Relative abundance indices are compared for breeding populations of 14 Massachusetts bird species. Indices from the top row to the bottom row of each matrix are BBS detection probability, BBS relative abundance from count data, and eBird detection probability from Generalized Linear Mixed Models and Random Forest models. Scatter plots and correlation coefficients are provided for each comparison and data distributions are given for each metric. Overall, the correspondence across indices was stronger between their annual estimates than their inter-annual changes. (A) Compares the correspondence between each index's annual estimates of relative abundance. (B) Compares the correspondence between each index's inter-annual changes in relative abundance.

* indicates the strength of a significant Pearson's correlation coefficient (or Spearman's correlation when data were not normally distributed).

S2 Fig 1. Correlation matrices of (A) annual estimates and (B) inter-annual changes of four relative abundance indices for Brown-headed Cowbird (*Molothrus ater*).

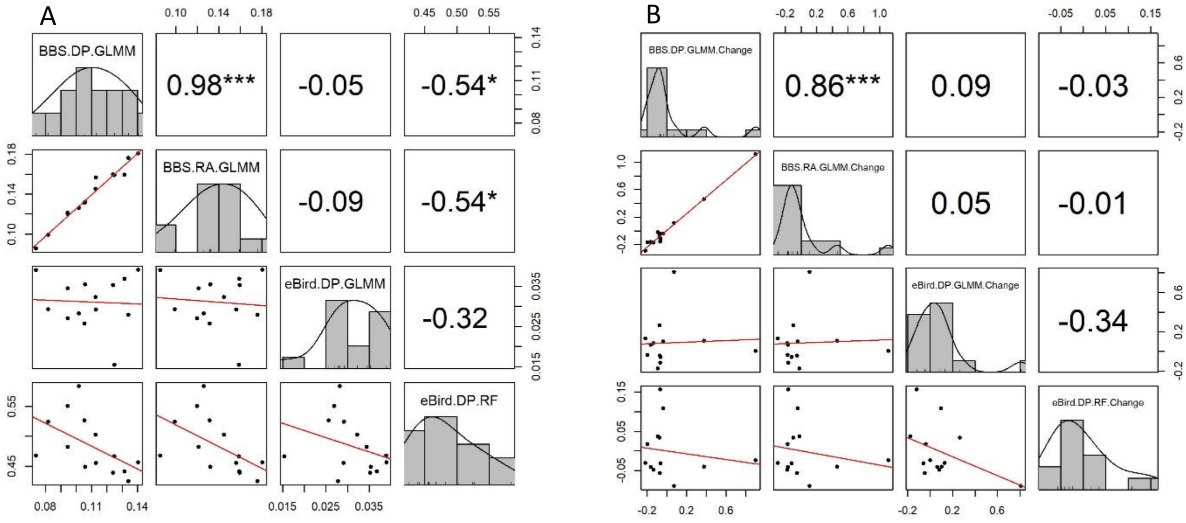

S2 Fig 2. Correlation matrices of (A) annual estimates and (B) inter-annual changes of four relative abundance indices for Common Grackle (*Quiscalus quiscula*).

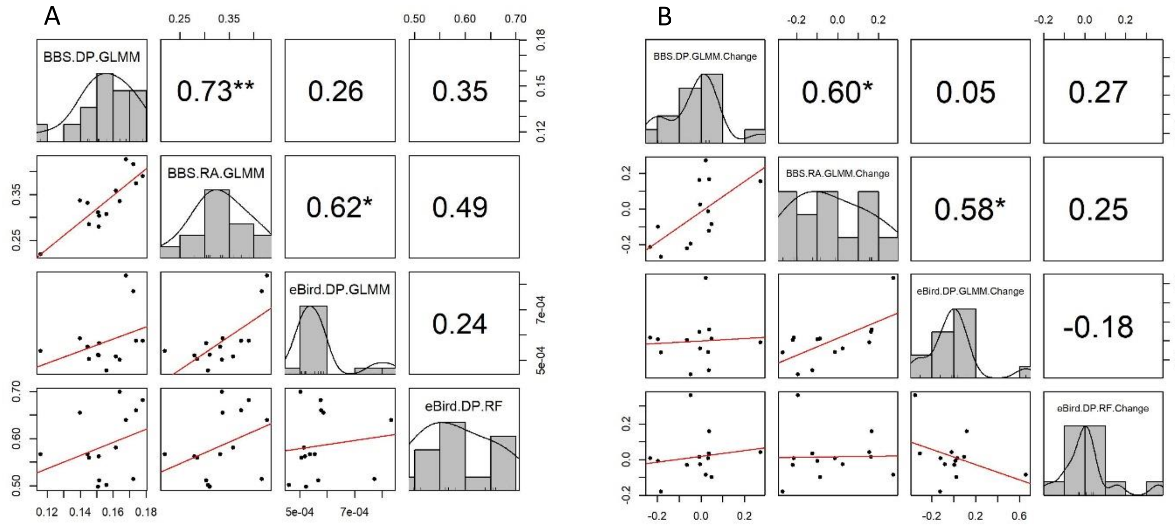

S2 Fig 3. Correlation matrices of (A) annual estimates and (B) inter-annual changes of four relative abundance indices for Downy Woodpecker (*Dryobates pubescens*).

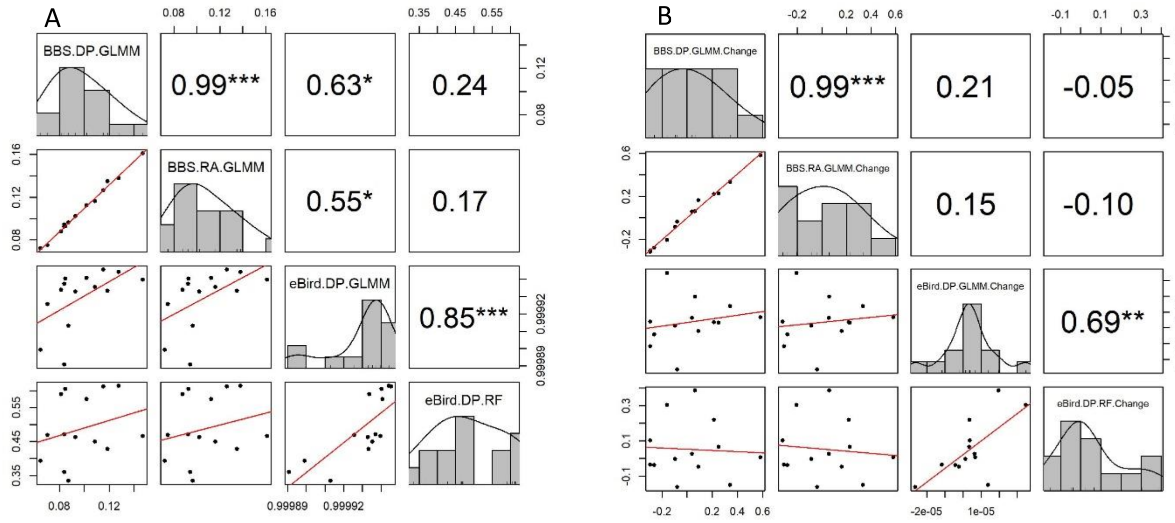

S2 Fig 4. Correlation matrices of (A) annual estimates and (B) inter-annual changes of four relative abundance indices for European Starling (*Sturnus vulgaris*).

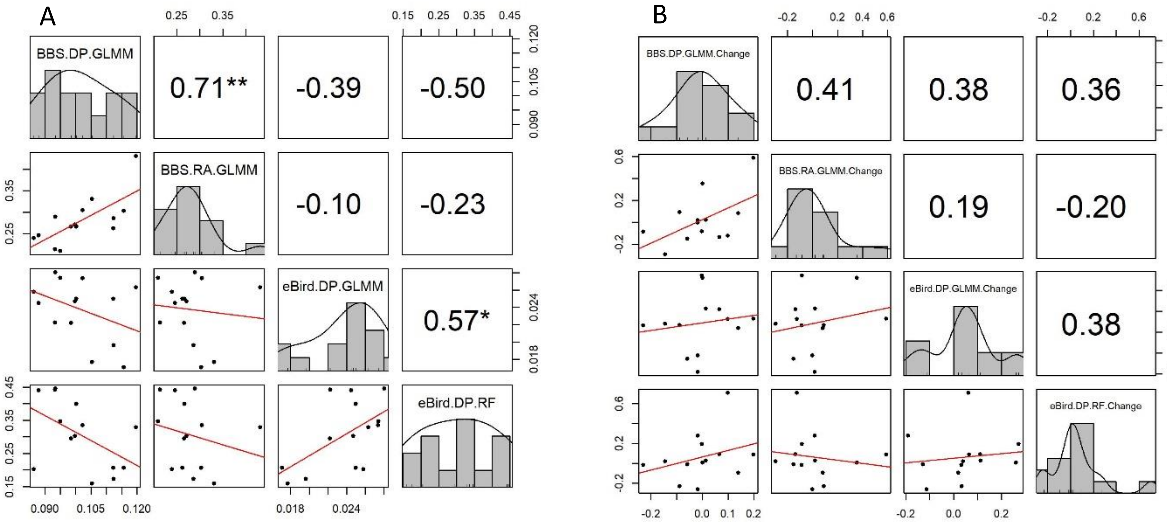

S2 Fig 5. Correlation matrices of (A) annual estimates and (B) inter-annual changes of four relative abundance indices for Hairy Woodpecker (*Dryobates villosus*).

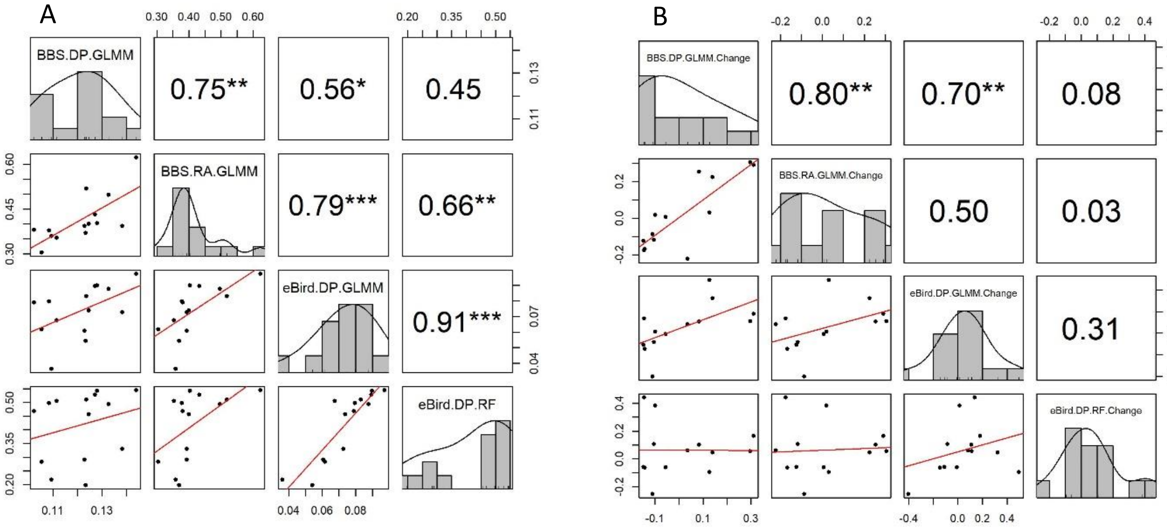

S2 Fig 6. Correlation matrices of (A) annual estimates and (B) inter-annual changes of four relative abundance indices for House Sparrow (*Passer domesticus*).

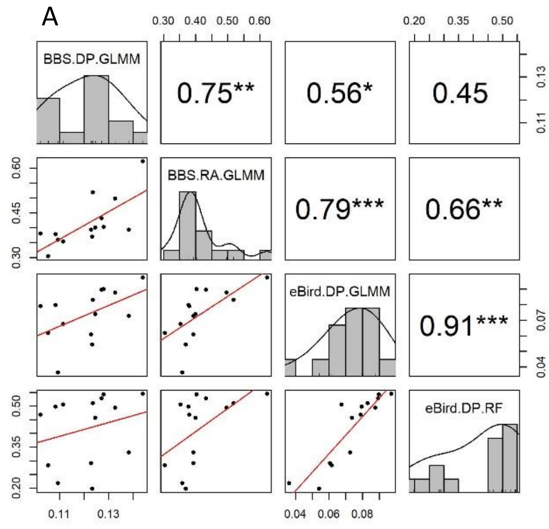

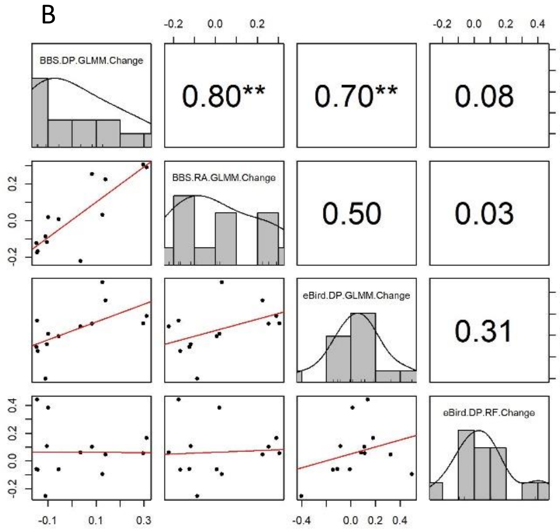

S2 Fig 7. Correlation matrices of (A) annual estimates and (B) inter-annual changes of four relative abundance indices for Mourning Dove (*Zenaida macroura*).

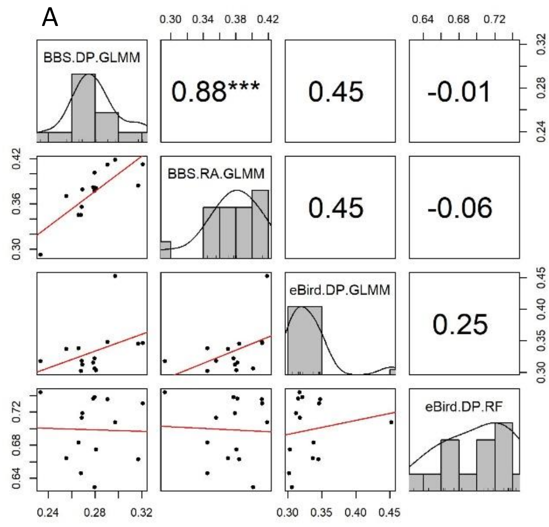

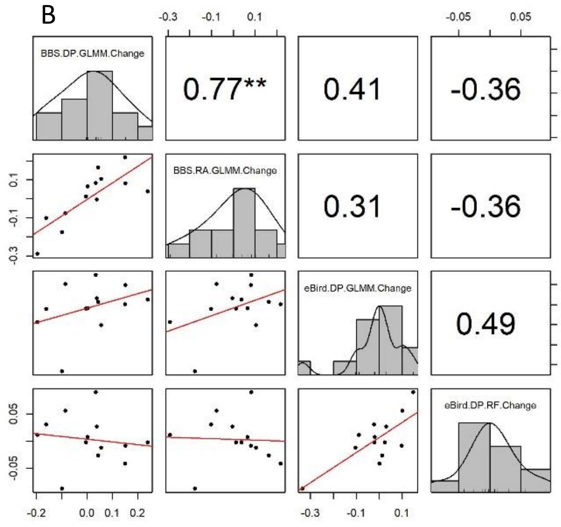

S2 Fig 8. Correlation matrices of (A) annual estimates and (B) inter-annual changes of four relative abundance indices for Northern Flicker (*Colaptes auratus*).

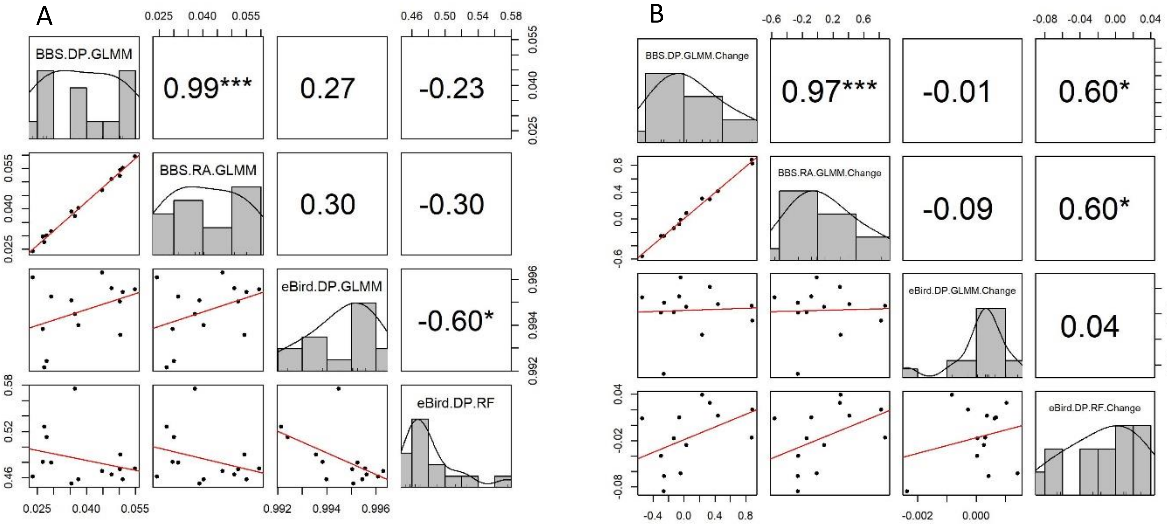

S2 Fig 9. Correlation matrices of (A) annual estimates and (B) inter-annual changes of four relative abundance indices for Osprey (*Pandion haliaetus*).

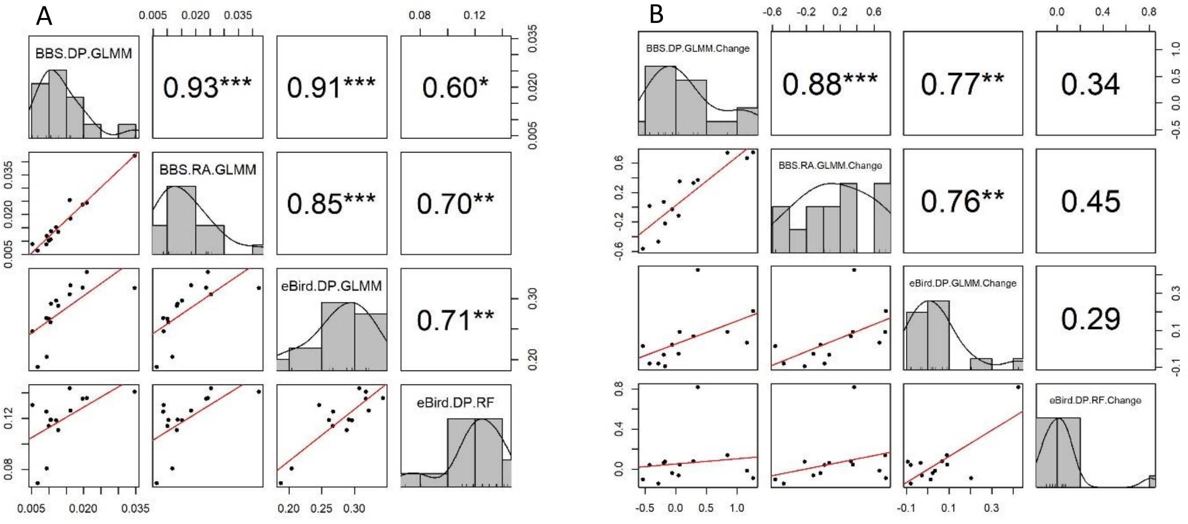

S2 Fig 10. Correlation matrices of (A) annual estimates and (B) inter-annual changes of four relative abundance indices for Pileated Woodpecker (*Dryocopus pileatus*).

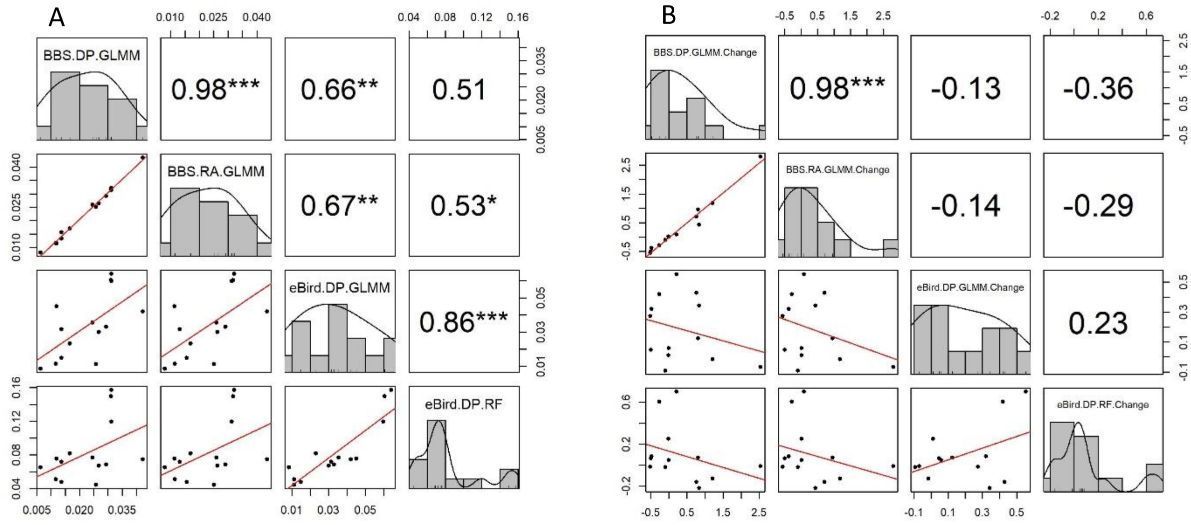

S2 Fig 11. Correlation matrices of (A) annual estimates and (B) inter-annual changes of four relative abundance indices for Red-bellied Woodpecker (*Melanerpes carolinus*).

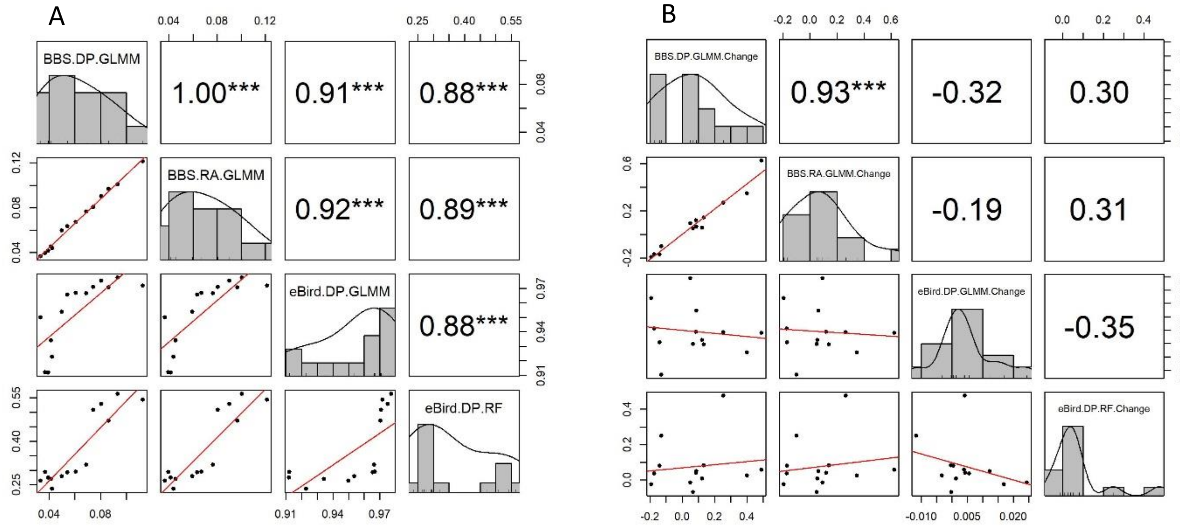

S2 Fig 12. Correlation matrices of (A) annual estimates and (B) inter-annual changes of four relative abundance indices for Red-tailed Hawk (*Buteo jamaicensis*).

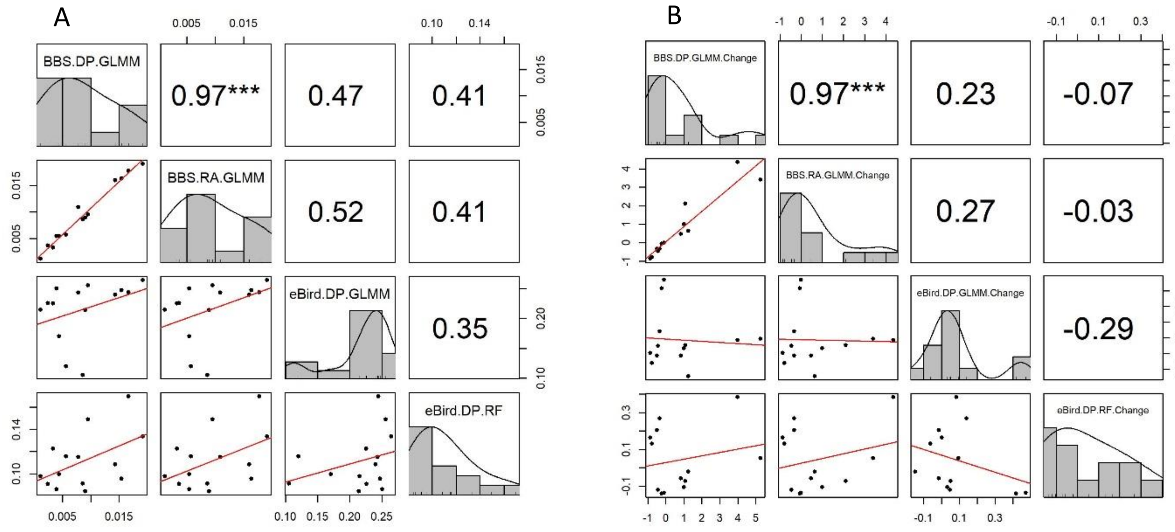

S2 Fig 13. Correlation matrices of (A) annual estimates and (B) inter-annual changes of four relative abundance indices for Red-winged Blackbird (*Agelaius phoeniceus*).

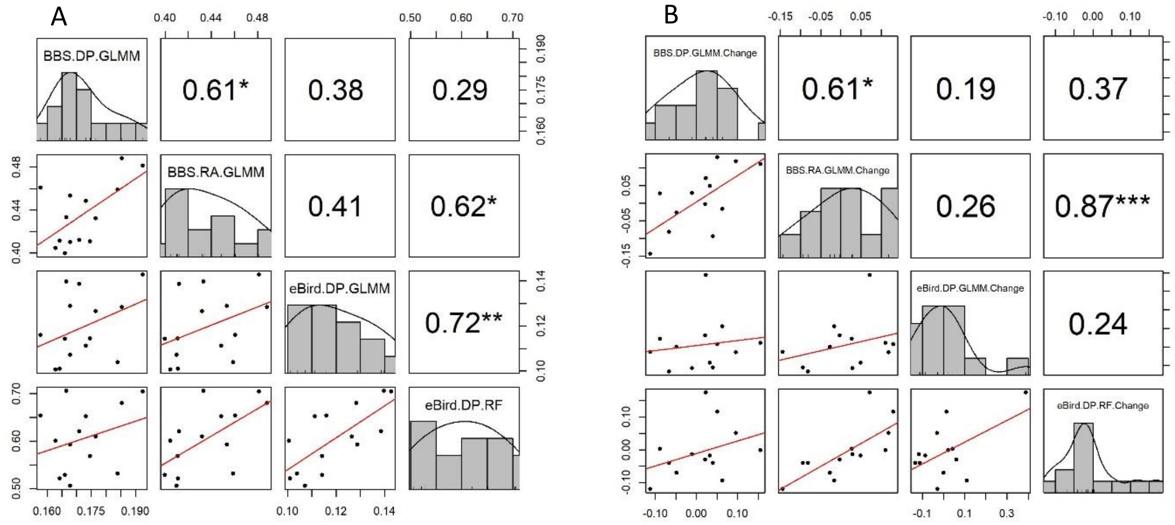

S2 Fig 14. Correlation matrices of (A) annual estimates and (B) inter-annual changes of four relative abundance indices for Turkey Vulture (*Cathartes aura*).

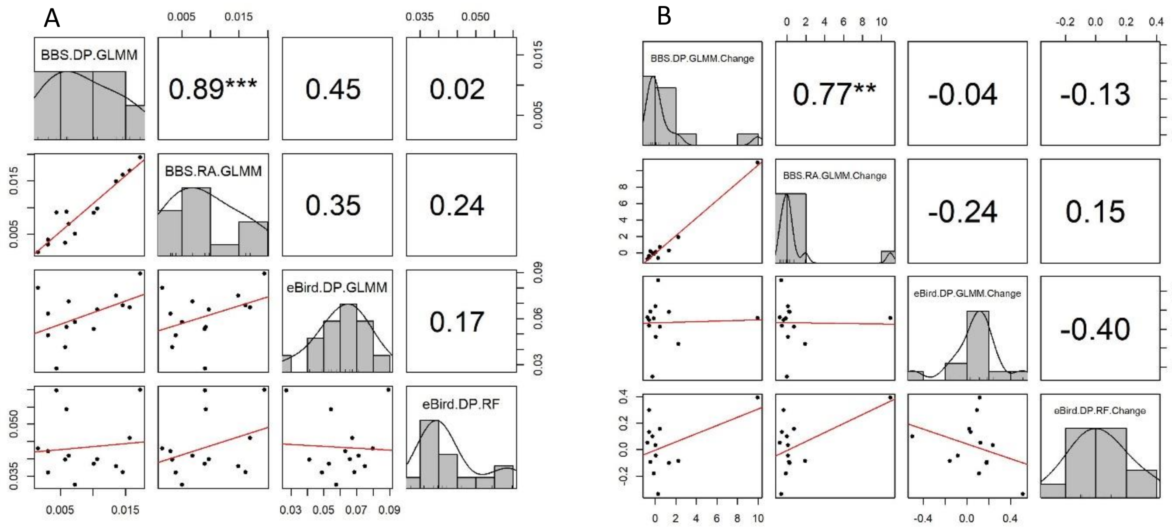

Supplement: S2 Appendix — Matrices for 14 Massachusetts bird species comparing the correspondence of annual estimates and their inter-annual changes between relative abundance indices. (PDF) [file pone.0257226.s002.pdf]
