# Supplementary material for: Comparing the reliability of relative bird abundance indices from standardized surveys and community science data at finer resolutions
Source: PLoS One. 2021 Sep 10;16(9):e0257226. doi: 10.1371/journal.pone.0257226 (PMC8432801; doi:10.1371/journal.pone.0257226)
Supplement: S3 Appendix — Multi-year trends (2005–2018) for 14 Massachusetts bird species. Trends are plotted from the annual estimates of each index smoothed by a LOESS curve and fit to generalized linear models. Time Series plots and linear model coefficients of the quadratic term of year for the annual estimates show the magnitude and directions of inter-annual changes for each index. (PDF) [file pone.0257226.s003.pdf]

S3 Appendix. **Multi-year trend and annual time series plots for four relative abundance indices.**

For 14 breeding bird species in Massachusetts, annual relative abundance estimates across different indices share similar directions in their multi-year trends (2005-2018). However, there are differences in the directions and magnitudes of the inter-annual changes for each index. (A) Multi-year trends for each relative abundance index calculated from the annual indices smoothed by a LOESS curve (solid lines) and fit to binomial generalized linear models (dashed lines). (B) Time Series plots of the annual estimates of each relative abundance index. S3 Table 1 shows the coefficients of the quadratic term for year in linear models of annual relative bird abundance estimates over time for these species. Relative abundance indices for each species vary between linear and curvilinear trends, with curvilinear trends being more common in eBird indices.

S3 Fig 1. (A) Multi-year trend and (B) annual time series plots of four relative abundance indices for Brown-headed Cowbird (*Molothrus ater*).

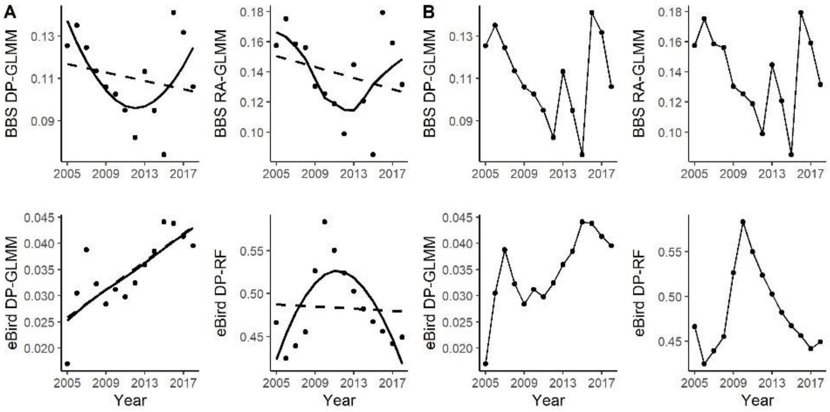

S3 Fig 2. (A) Multi-year trend and (B) annual time series plots of four relative abundance indices for Common Grackle (*Quiscalus quiscula*).

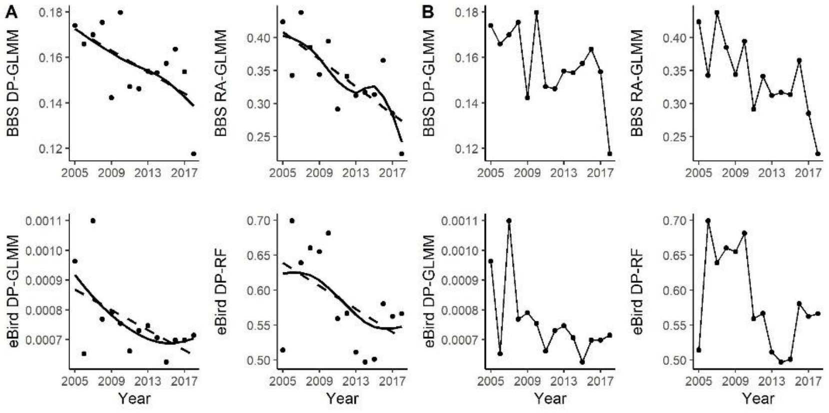

S3 Fig 3. (A) Multi-year trend and (B) annual time series plots of four relative abundance indices for Downy Woodpecker (*Dryobates pubescens*).

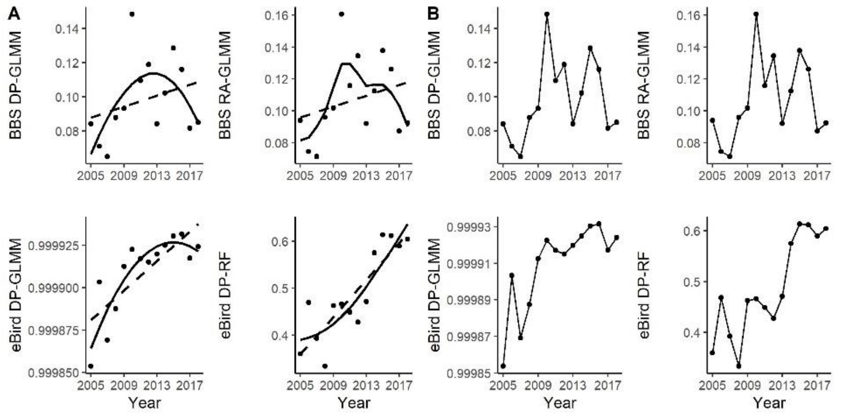

S3 Fig 4. (A) Multi-year trend and (B) annual time series plots of four relative abundance indices for European Starling (*Sturnus vulgaris*).

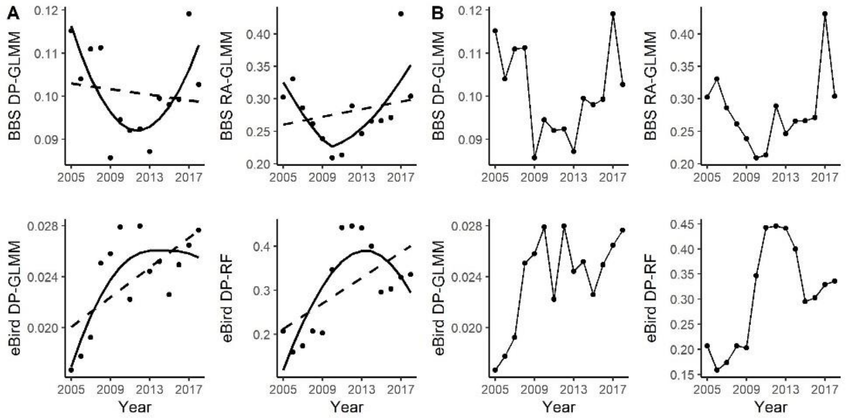

S3 Fig 5. (A) Multi-year trend and (B) annual time series plots of four relative abundance indices for Hairy Woodpecker (*Dryobates villosus*).

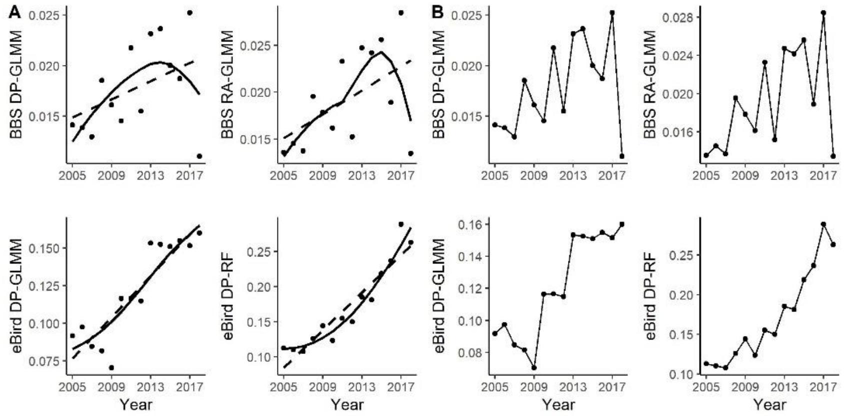

S3 Fig 6. (A) Multi-year trend and (B) annual time series plots of four relative abundance indices for House Sparrow (*Passer domesticus*).

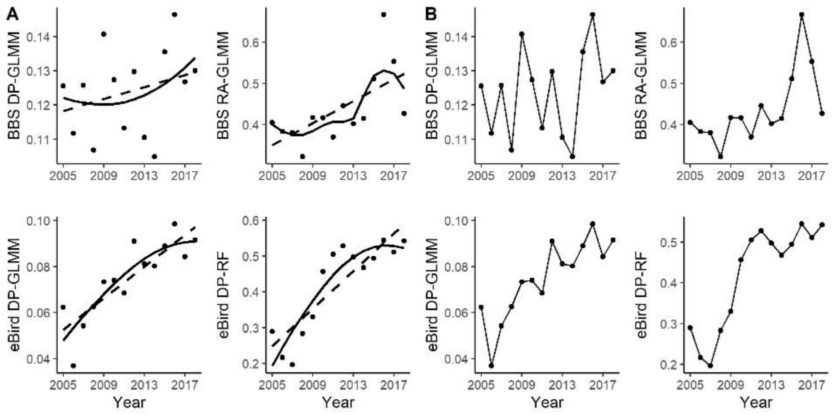

S3 Fig 7. (A) Multi-year trend and (B) annual time series plots of four relative abundance indices for Mourning Dove (*Zenaida macroura*).

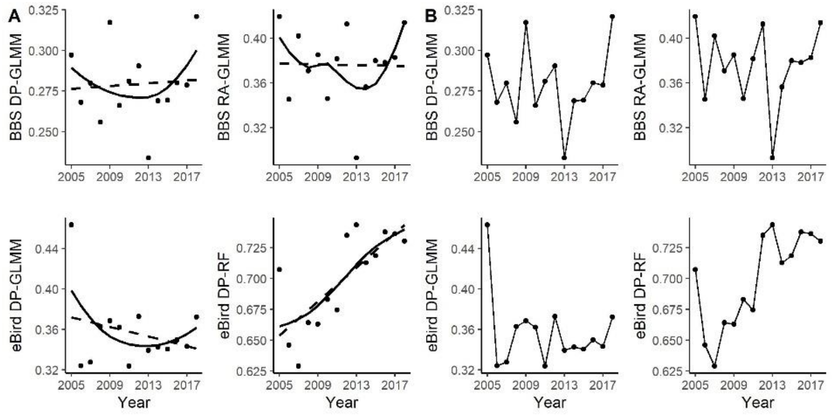

S3 Fig 8. (A) Multi-year trend and (B) annual time series plots of four relative abundance indices for Northern Flicker (*Colaptes auratus*).

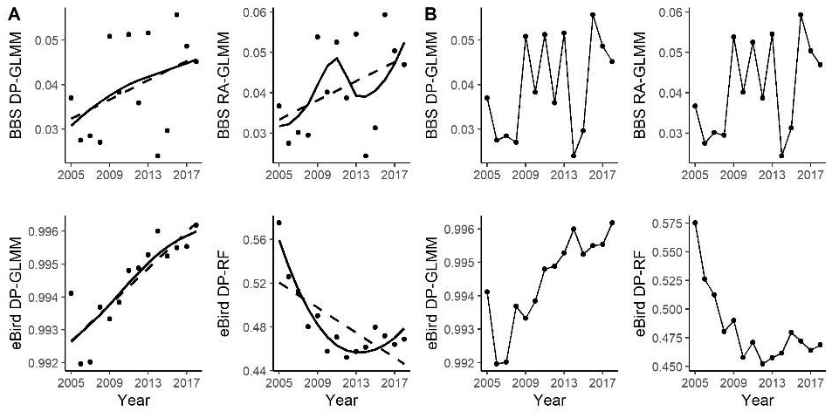

S3 Fig 9. (A) Multi-year trend and (B) annual time series plots of four relative abundance indices for Osprey (*Pandion haliaetus*).

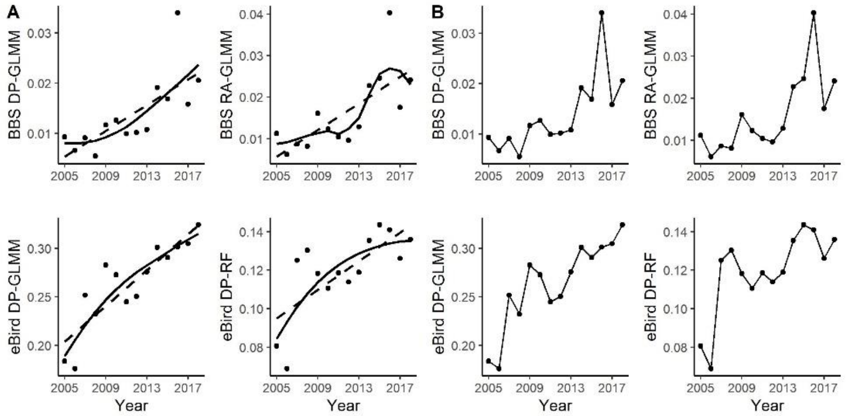

S3 Fig 10. (A) Multi-year trend and (B) annual time series plots of four relative abundance indices for Pileated Woodpecker (*Dryocopus pileatus*).

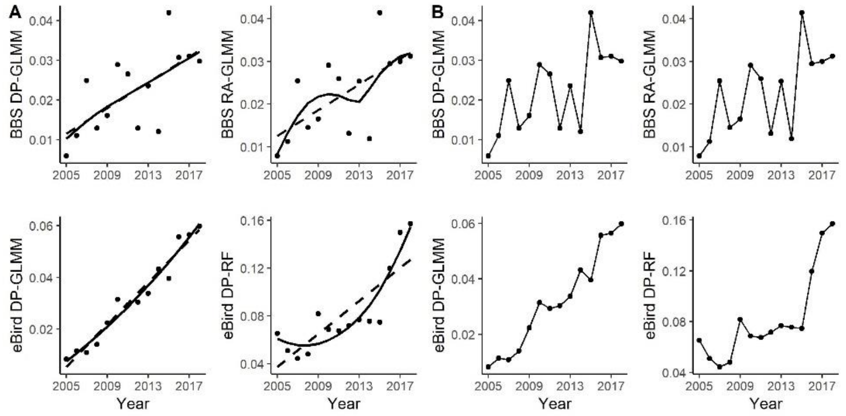

S3 Fig 11. (A) Multi-year trend and (B) annual time series plots of four relative abundance indices for Red-bellied Woodpecker (*Melanerpes carolinus*).

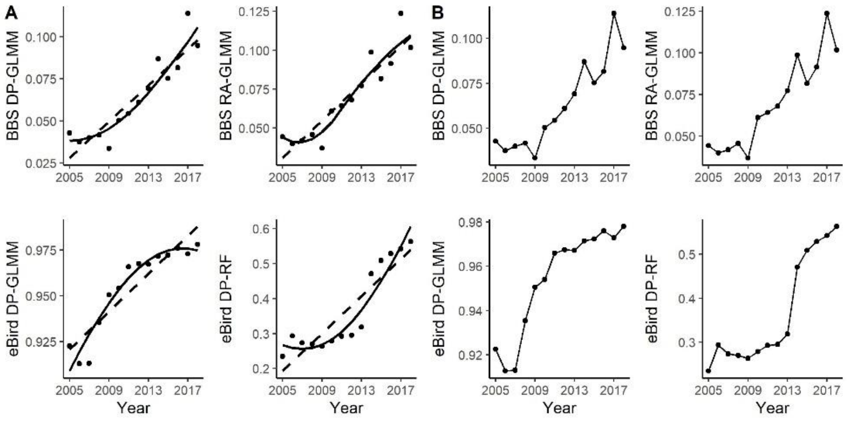

S3 Fig 12. (A) Multi-year trend and (B) annual time series plots of four relative abundance indices for Red-tailed Hawk (*Buteo jamaicensis*).

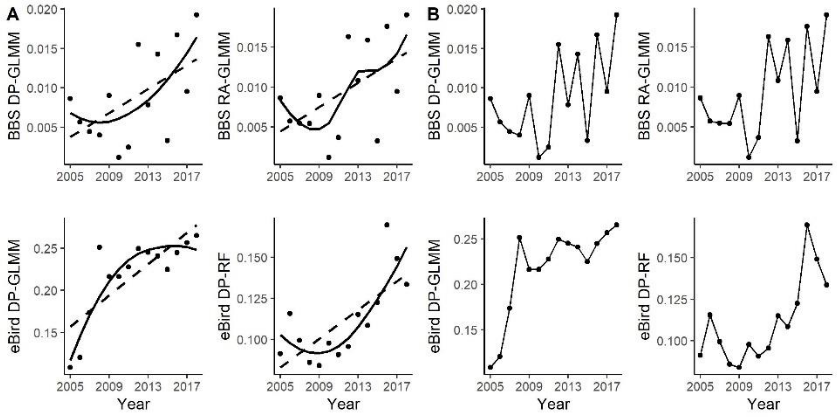

S3 Fig 13. (A) Multi-year trend and (B) annual time series plots of four relative abundance indices for Red-winged Blackbird (*Agelaius phoeniceus*).

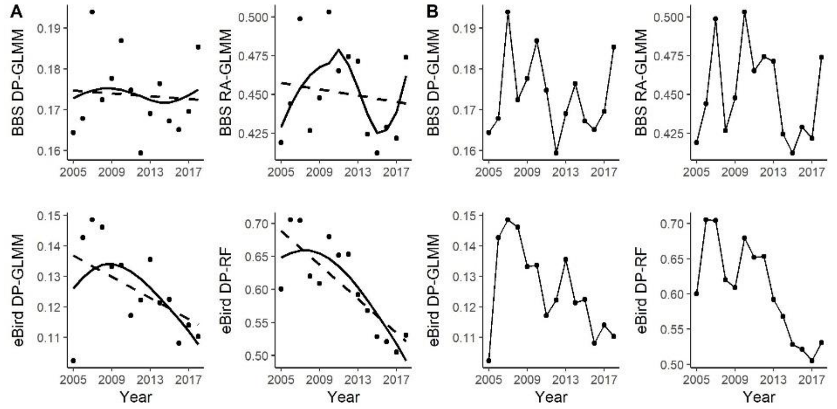

S3 Fig 14. (A) Multi-year trend and (B) annual time series plots of four relative abundance indices for Turkey Vulture (*Cathartes aura*).

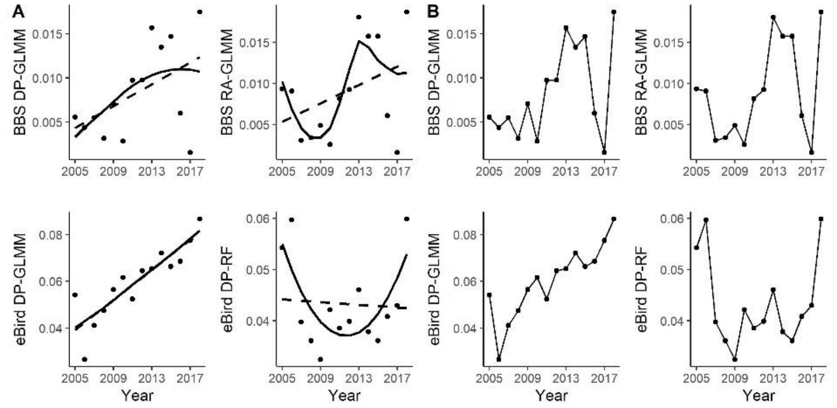

S3 Table 1. Linearity of multi-year bird abundance trends from 2005-2018 for four indices of relative abundance.

Family	Species	BBS DP	BBS RA	eBird DP-GLMM	eBird DP-RF
Icteridae	*Q. quiscula*	-0.01 (-0.04, 0.03)	0.00 (-0.03, 0.03)	0.02 (-0.01, 0.05)	0.00 (-0.04, 0.04)
	A. phoeniceus	0.00 (-0.04, 0.05)	-0.02 (-0.06, 0.02)	-0.02 (-0.05, 0.01)	-0.02 (-0.04, 0.00)
	M. ater	**0.04 (0.00, 0.07)**	**0.03 (0.00, 0.07)**	0.00 (-0.03, 0.02)	**-0.05 (-0.08, -0.02)**
Picidae	*C. auratus*	0.00 (-0.04, 0.04)	-0.01 (-0.05, 0.03)	0.00 (-0.03, 0.02)	**0.04 (0.02, 0.06)**
	D. villosus	-0.03 (-0.06, 0.01)	-0.02 (-0.06, 0.01)	0.00 (-0.02, 0.02)	**0.02 (0.01, 0.03)**
	D. pubescens	**-0.04 (-0.07, 0.00)**	**-0.04 (-0.07, 0.00)**	**-0.03 (-0.05, 0.01)**	0.01 (-0.01, 0.03)
	D. pileatus	0.00 (-0.04, 0.03)	0.00 (-0.04, 0.03)	0.01 (0.00, 0.01)	**0.03 (0.01, 0.05)**
	M. carolinus	0.01 (0.00, 0.03)	0.01 (-0.01, 0.02)	**-0.02 (-0.03, -0.01)**	**0.02 (0.01, 0.03)**
Accipitridae	*B. jamaicensis*	0.02 (-0.01, 0.05)	0.01 (-0.02, 0.05)	**-0.03 (-0.05, -0.01)**	**0.03 (0.00, 0.05)**
Pandionidae	*P. haliaetus*	0.01 (-0.02, 0.04)	0.01 (-0.02, 0.04)	-0.01 (-0.03, 0.01)	-0.02 (-0.05, 0.01)
Cathartidae	*C. aura*	-0.01 (-0.05, 0.03)	0.00 (-0.04, 0.05)	-0.02 (-0.06, 0.02)	**0.03 (0.01, 0.06)**
Columbidae	*Z. macroura*	0.03 (-0.01, 0.07)	0.03 (-0.01, 0.07)	0.03 (-0.01, 0.07)	0.00 (-0.03, 0.03)
Sturnidae	*S. vulgaris*	-0.01 (-0.05, 0.03)	0.00 (-0.04, 0.04)	0.00 (-0.02, 0.02)	**-0.05 (-0.02, -0.08)**
Passeridae	*P. domesticus*	0.01 (-0.03, 0.05)	0.01 (-0.02, 0.04)	-0.01 (-0.03, 0.01)	-0.02 (-0.04, 0.00)

Coefficients and their 95th percent confidence intervals of the quadratic term for year in linear models of annual relative bird abundance estimates over time for 14 Massachusetts bird species. The sign and magnitude of the coefficients describes the curve of non-linear trends. Positive values indicate convex curved trends, negative values indicate concave curved trends, and zero indicates a linear trend. Non-zero (curvilinear trends) are bolded and are found predominantly in the eBird indices. Overall, relative abundance indices for each species vary between linear and curvilinear trends. Woodpeckers as a taxonomic group have relatively linear trends.

DP= Detection Probability

RA= Relative Abundance (from BBS count data)
