# Supplementary material for: Comparing the reliability of relative bird abundance indices from standardized surveys and community science data at finer resolutions
Source: PLoS One. 2021 Sep 10;16(9):e0257226. doi: 10.1371/journal.pone.0257226 (PMC8432801; doi:10.1371/journal.pone.0257226)

S4 Appendix. **Correlation between annual relative abundance indices and their inter-annual trends derived from two datasets across Massachusetts towns for 14 bird species.**

Weighted Pearson's correlation coefficients of town-level relative abundance indices between BBS detection probability and two eBird modeling methods **A, B)** eBird detection probability from Generalized Linear Mixed Models (GLMM) and **C, D)** eBird detection probability from Random Forests (RF) mapped by towns to show the correspondence of these indices across Massachusetts. Only towns with both BBS and eBird surveys (maximum of 25 towns) and that contain records of the species are mapped. Left-sided maps **(A, D)** compare the annual estimates of these indices and the right-sided maps **(B, D)** compare their inter-annual trends.

S4 Fig 1. Correlation coefficients between town-level BBS and eBird detection probability indices across Massachusetts for Brown-headed Cowbird (*Molothrus ater*). (A, B) compare BBS with eBird GLMM methods, and (C, D) compare BBS with eBird RF methods. Left-sided maps (A, C) compare annual estimates and right-sided maps compare inter-annual changes in these estimates.

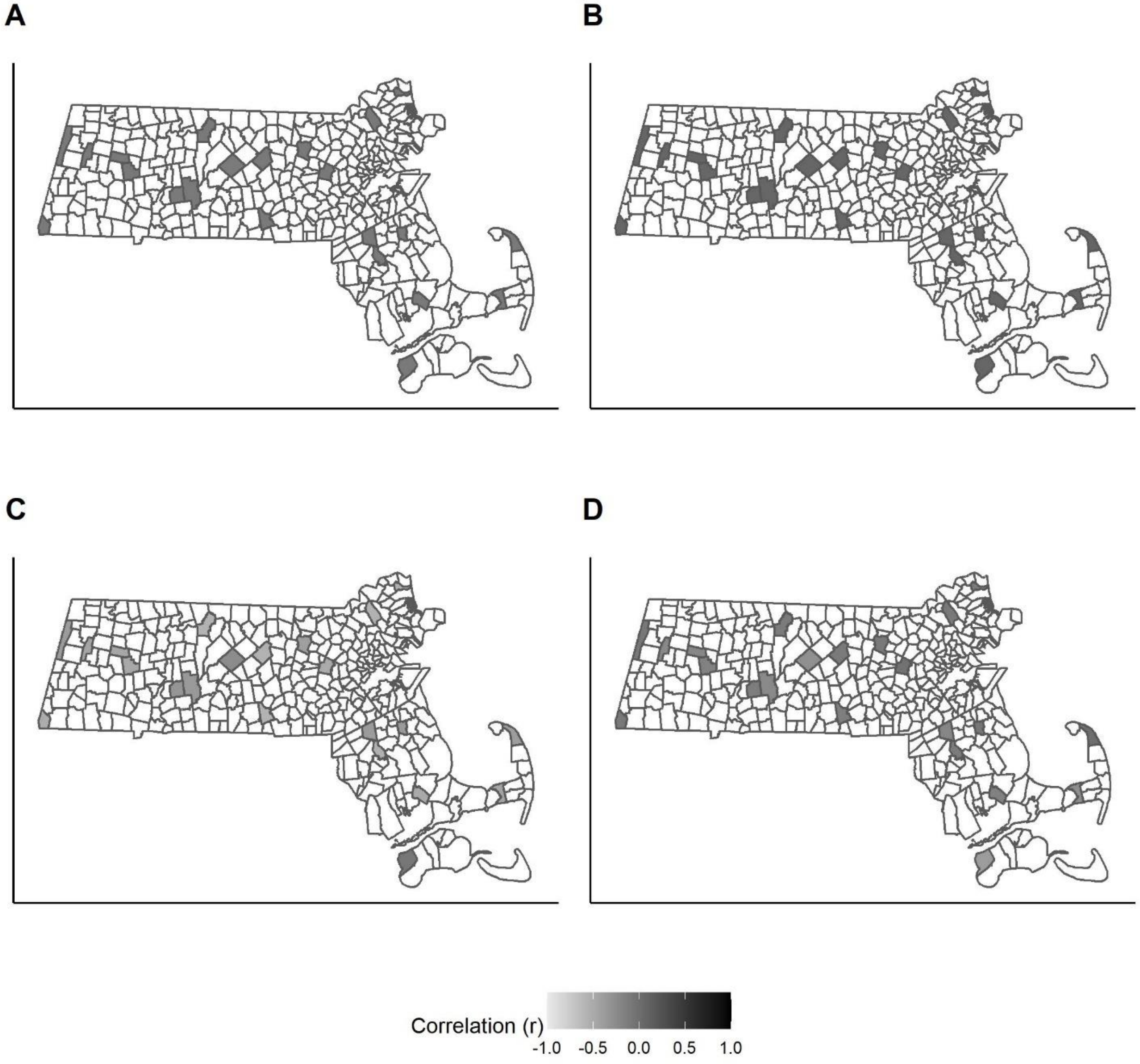

S4 Fig 2. Correlation coefficients between town-level BBS and eBird detection probability indices across Massachusetts for Common Grackle (*Quiscalus quiscula*). (A, B) compare BBS with eBird GLMM methods, and (C, D) compare BBS with eBird RF methods. Left-sided maps (A, C) compare annual estimates and right-sided maps compare inter-annual changes in these estimates.

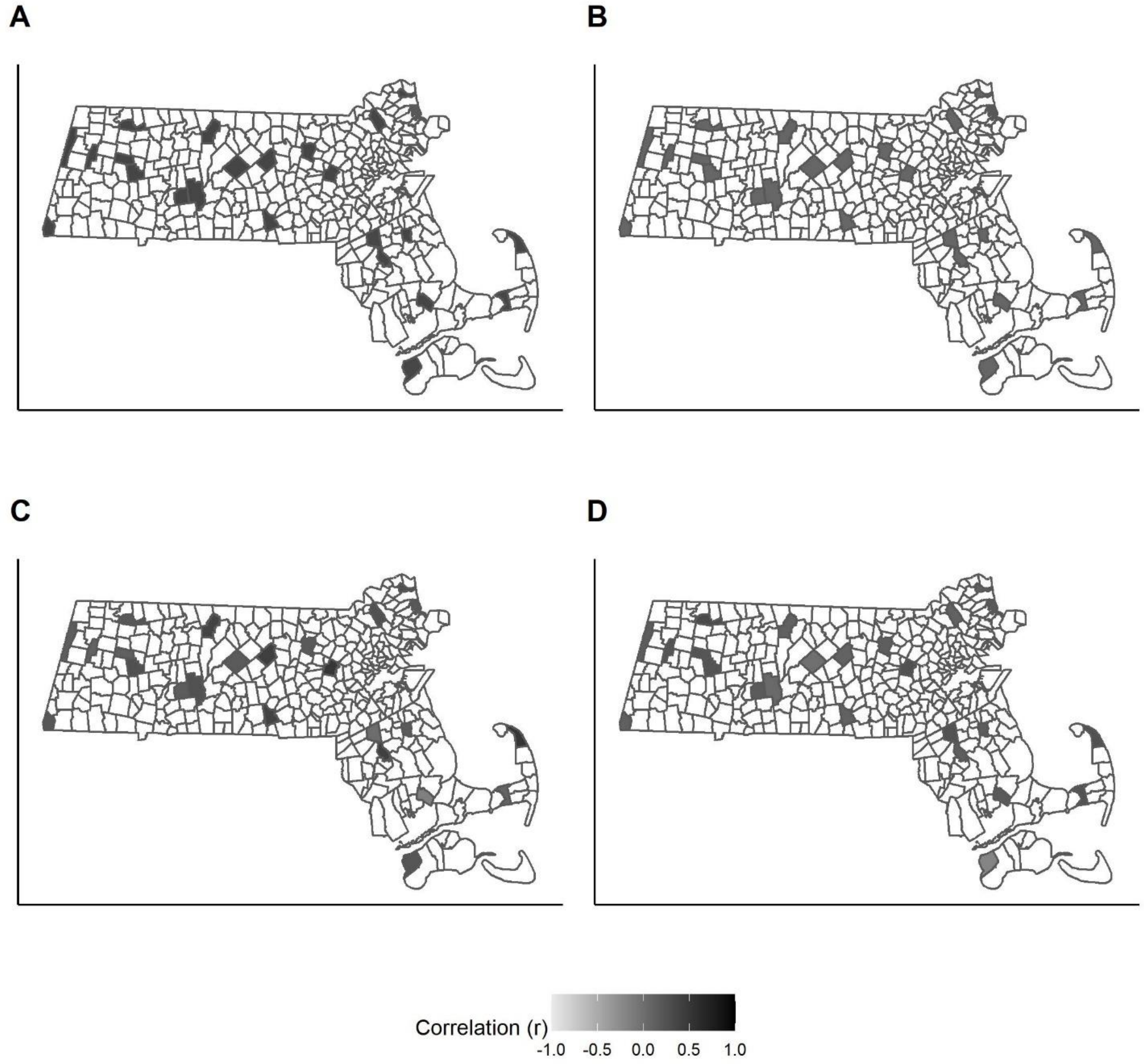

S4 Fig 3. Correlation coefficients between town-level BBS and eBird detection probability indices across Massachusetts for Downy Woodpecker (*Dryobates pubescens*). (A, B) compare BBS with eBird GLMM methods, and (C, D) compare BBS with eBird RF methods. Left-sided maps (A, C) compare annual estimates and right-sided maps compare inter-annual changes in these estimates.

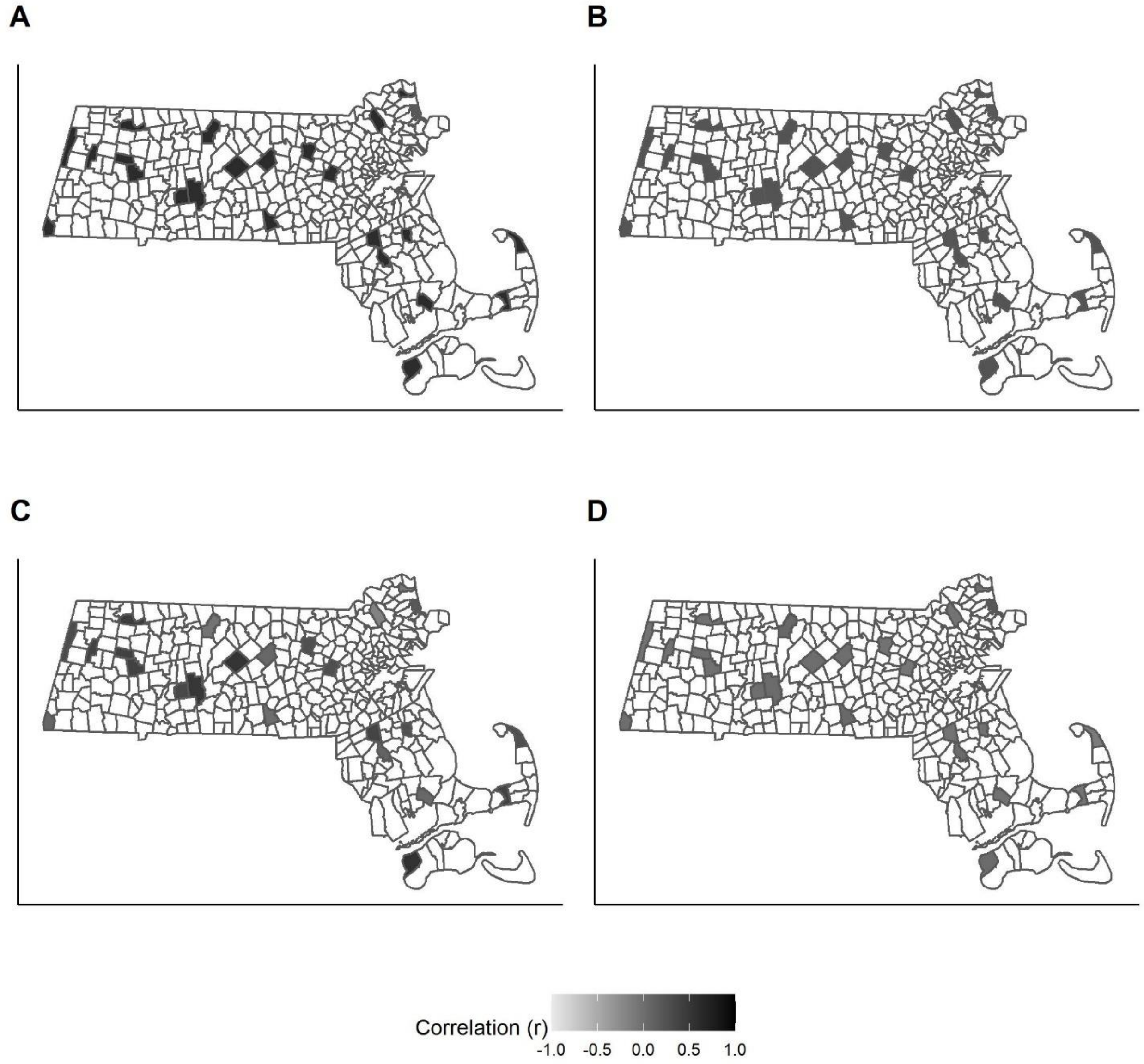

S4 Fig 4. Correlation coefficients between town-level BBS and eBird detection probability indices across Massachusetts for European Starling (*Sturnus vulgaris*). (A, B) compare BBS with eBird GLMM methods, and (C, D) compare BBS with eBird RF methods. Left-sided maps (A, C) compare annual estimates and right-sided maps compare inter-annual changes in these estimates.

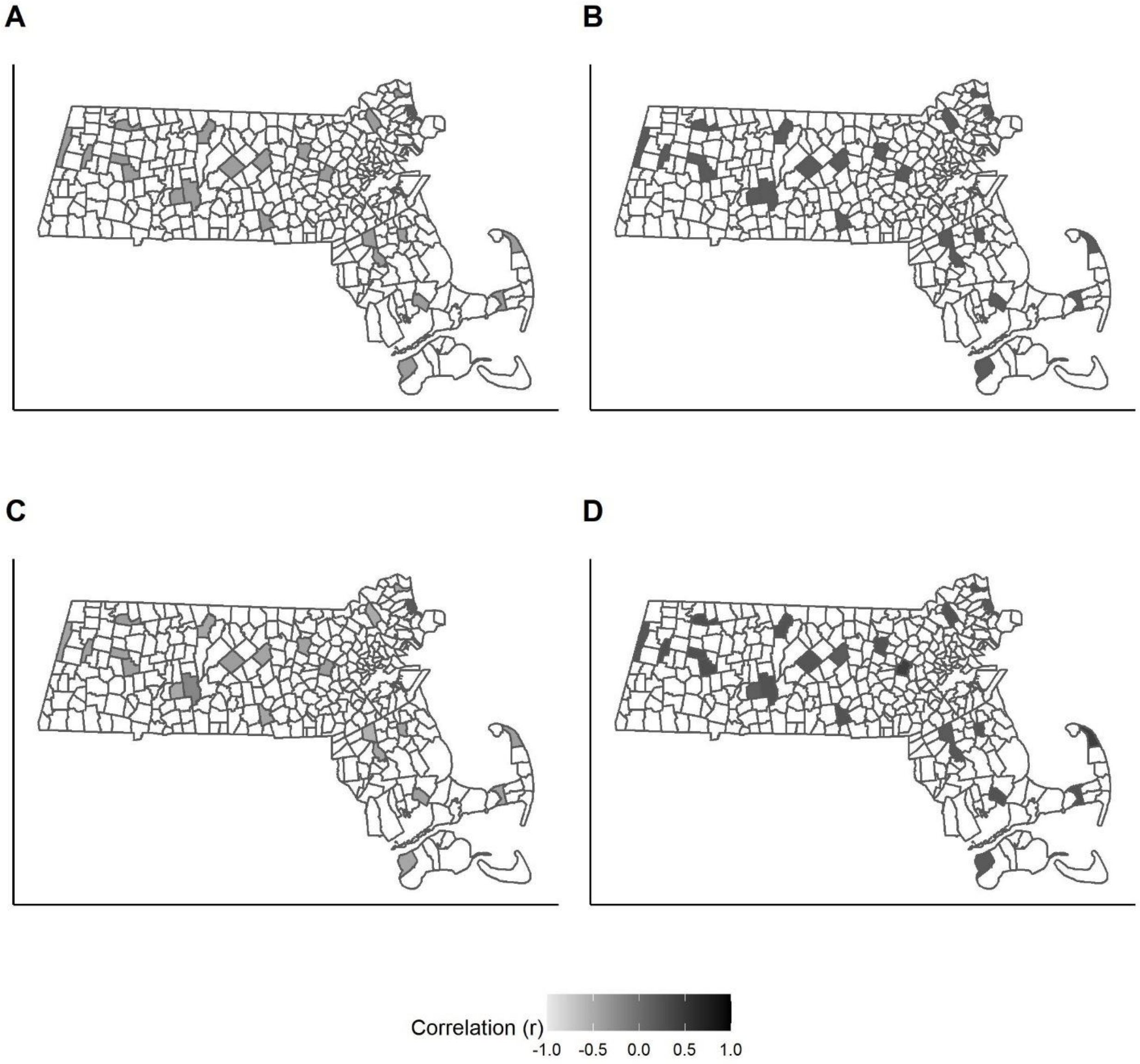

S4 Fig 5. Correlation coefficients between town-level BBS and eBird detection probability indices across Massachusetts for Hairy Woodpecker (*Dryobates villosus*). (A, B) compare BBS with eBird GLMM methods, and (C, D) compare BBS with eBird RF methods. Left-sided maps (A, C) compare annual estimates and right-sided maps compare inter-annual changes in these estimates.

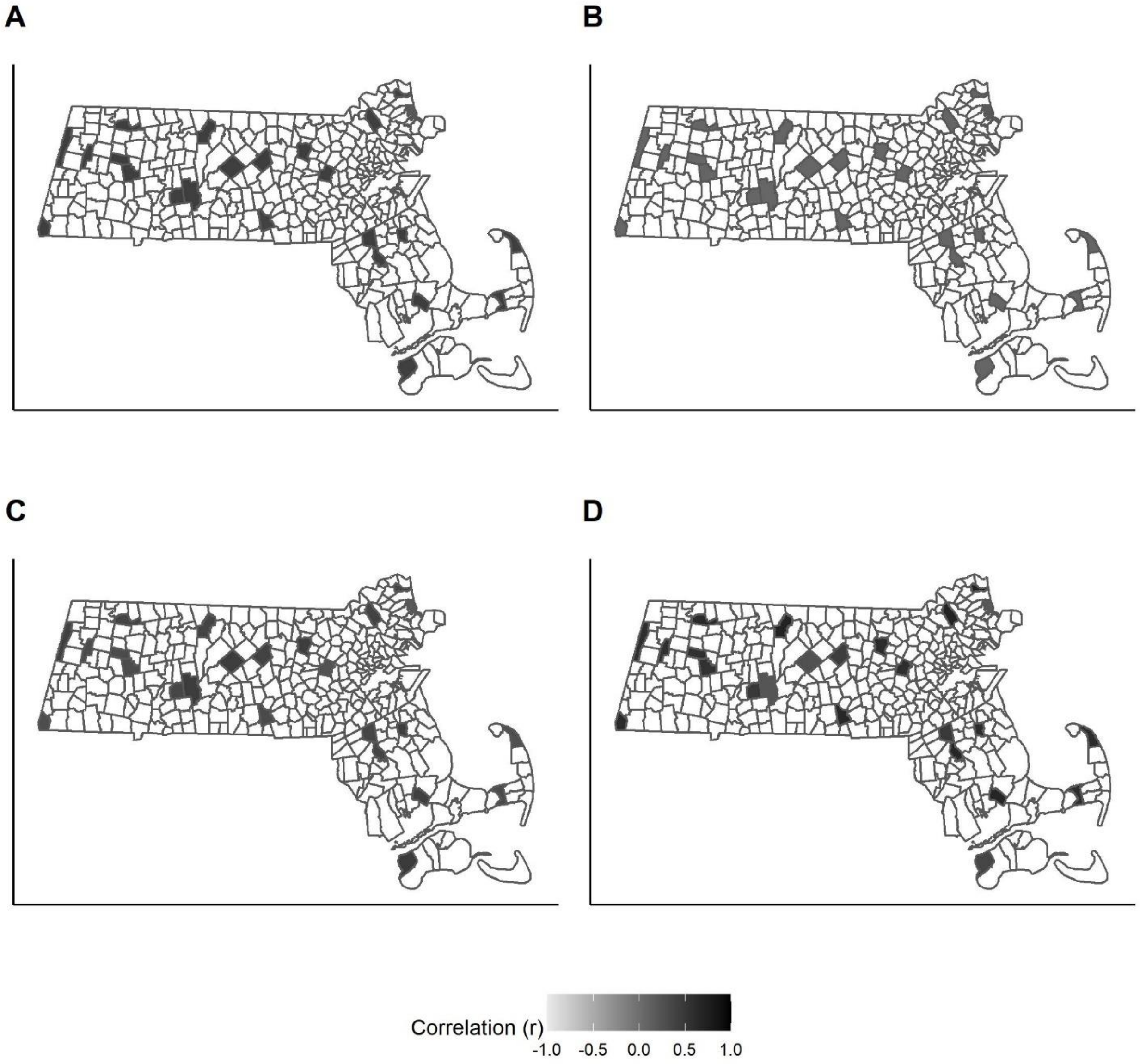

S4 Fig 6. Correlation coefficients between town-level BBS and eBird detection probability indices across Massachusetts for House Sparrow (*Passer domesticus*). (A, B) compare BBS with eBird GLMM methods, and (C, D) compare BBS with eBird RF methods. Left-sided maps (A, C) compare annual estimates and right-sided maps compare inter-annual changes in these estimates.

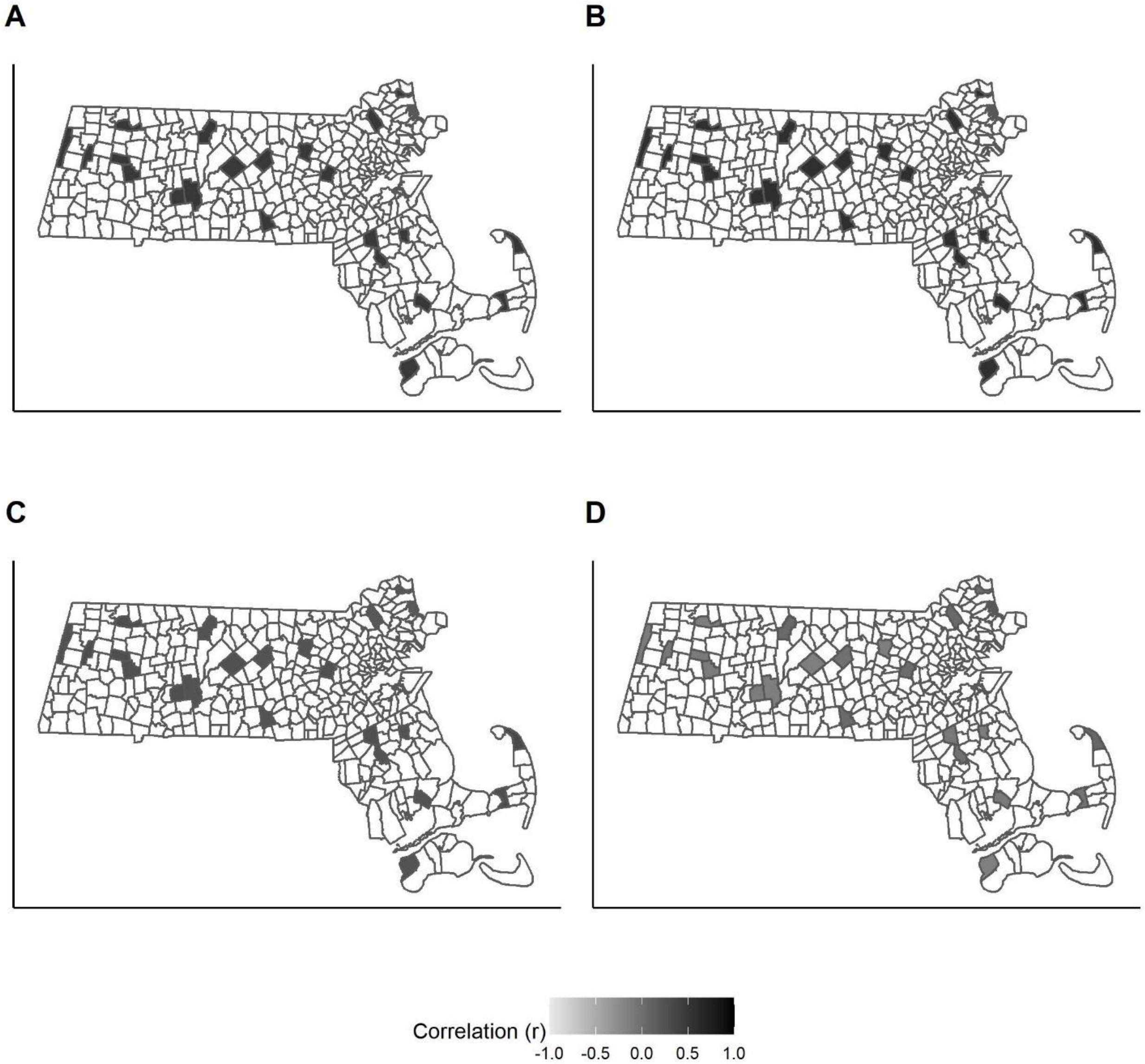

S4 Fig 7. Correlation coefficients between town-level BBS and eBird detection probability indices across Massachusetts for Mourning Dove (*Zenaida macroura*). (A, B) compare BBS with eBird GLMM methods, and (C, D) compare BBS with eBird RF methods. Left-sided maps (A, C) compare annual estimates and right-sided maps compare inter-annual changes in these estimates.

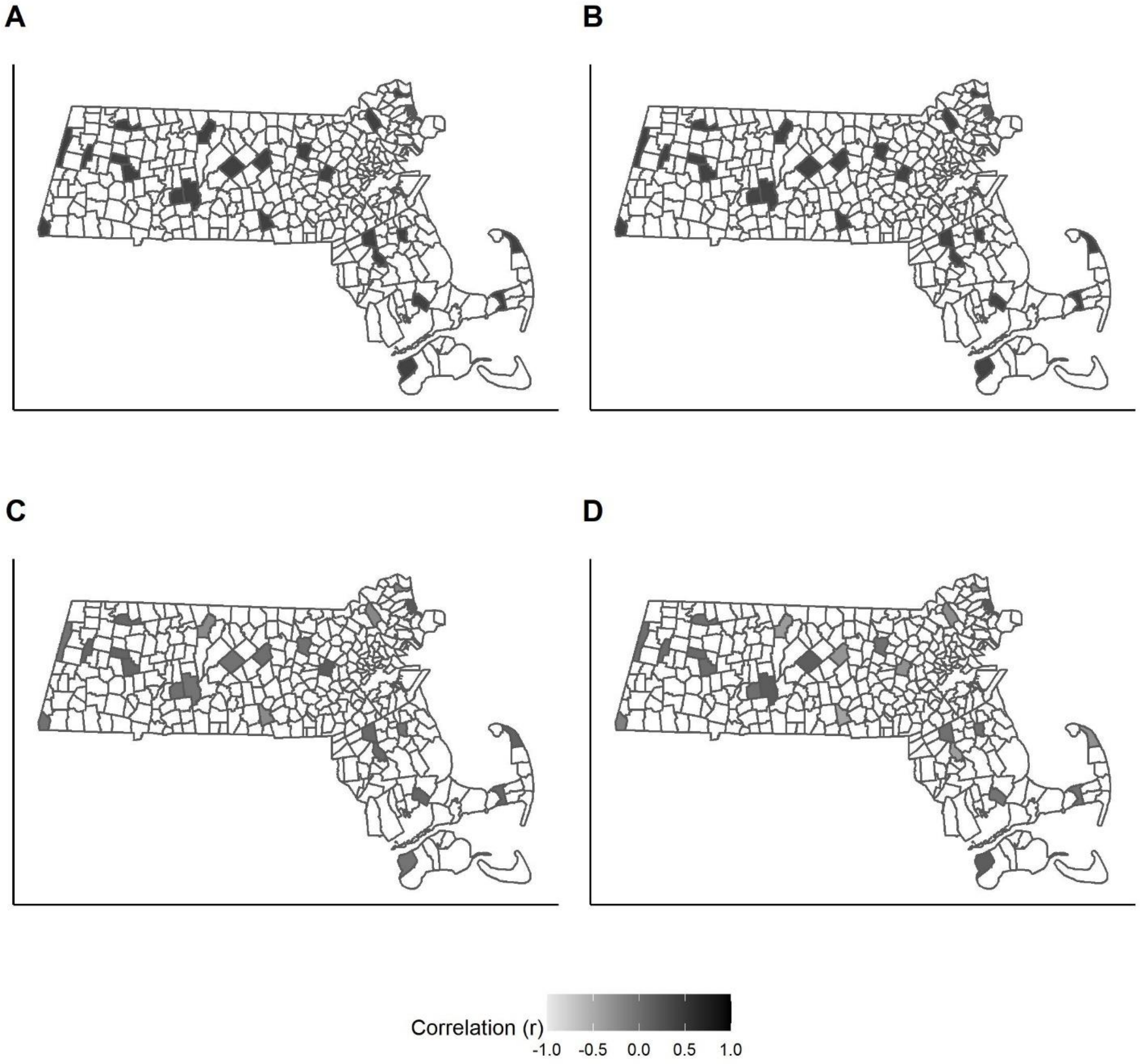

S4 Fig 8. Correlation coefficients between town-level BBS and eBird detection probability indices across Massachusetts for Northern Flicker (*Colaptes auratus*). (A, B) compare BBS with eBird GLMM methods, and (C, D) compare BBS with eBird RF methods. Left-sided maps (A, C) compare annual estimates and right-sided maps compare inter-annual changes in these estimates.

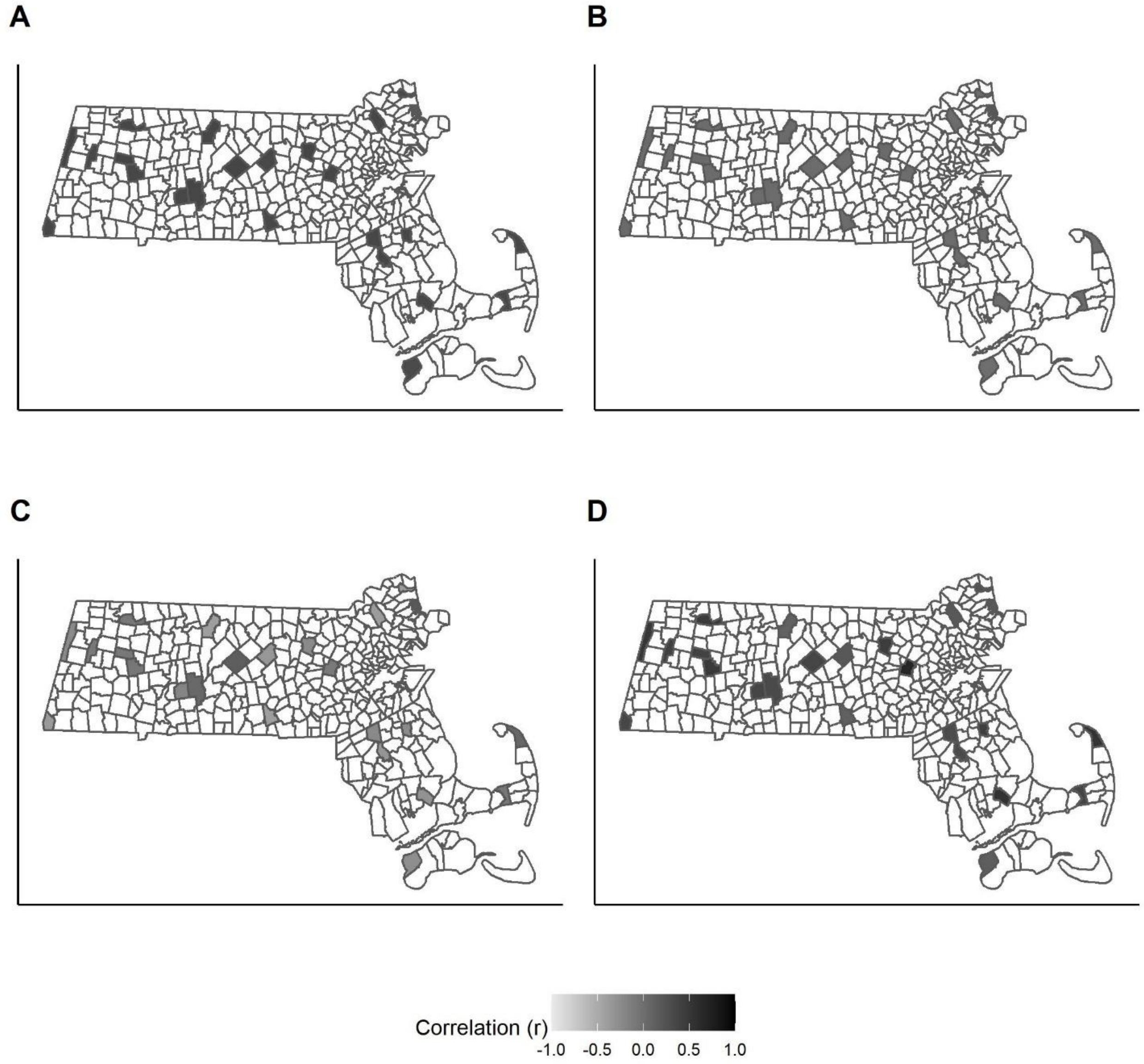

S4 Fig 9. Correlation coefficients between town-level BBS and eBird detection probability indices across Massachusetts for Osprey (*Pandion haliaetus*). (A, B) compare BBS with eBird GLMM methods, and (C, D) compare BBS with eBird RF methods. Left-sided maps (A, C) compare annual estimates and right-sided maps compare inter-annual changes in these estimates.

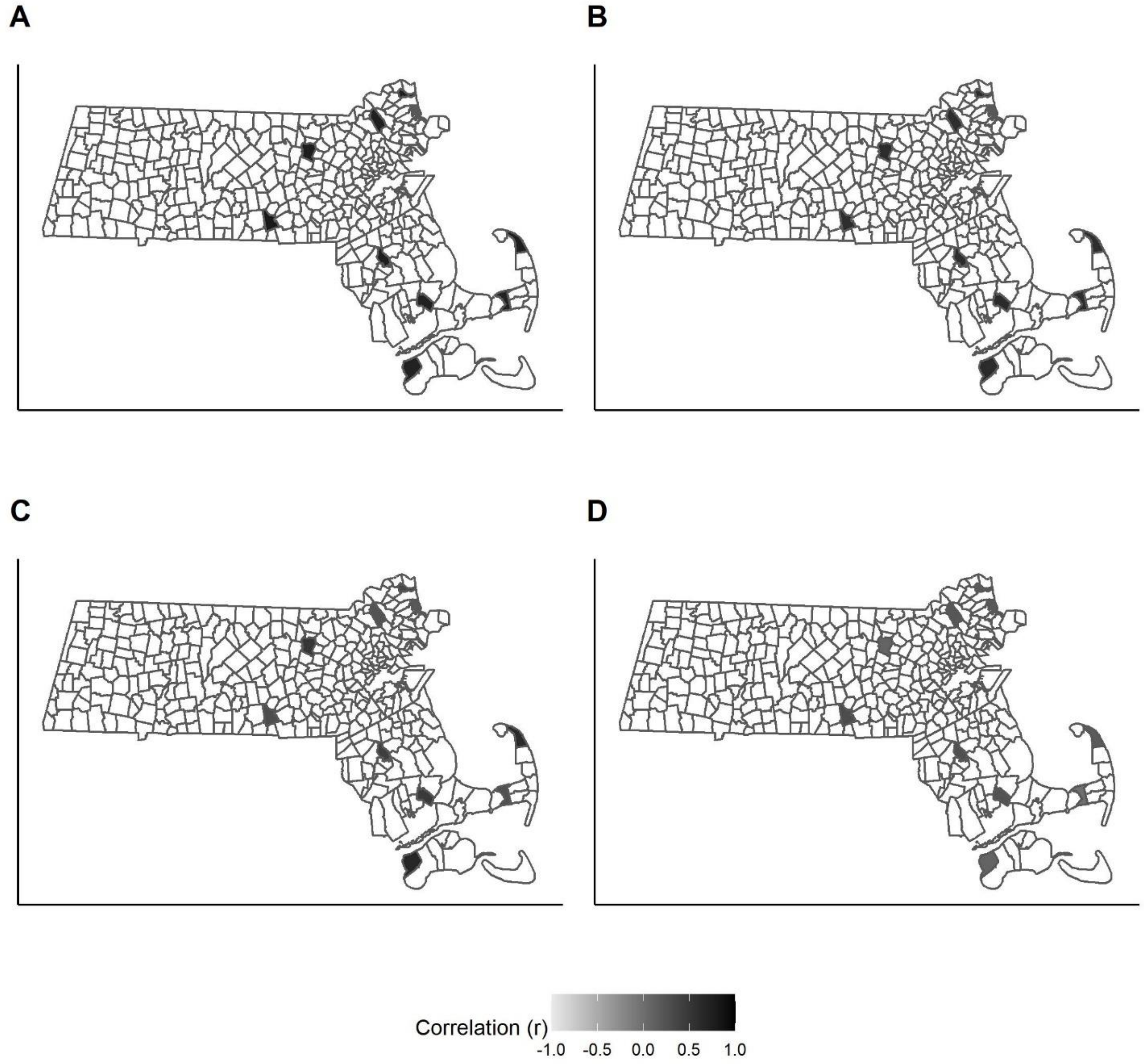

S4 Fig 10. Correlation coefficients between town-level BBS and eBird detection probability indices across Massachusetts for Pileated Woodpecker (*Dryocopus pileatus*). (A, B) compare BBS with eBird GLMM methods, and (C, D) compare BBS with eBird RF methods. Left-sided maps (A, C) compare annual estimates and right-sided maps compare inter-annual changes in these estimates.

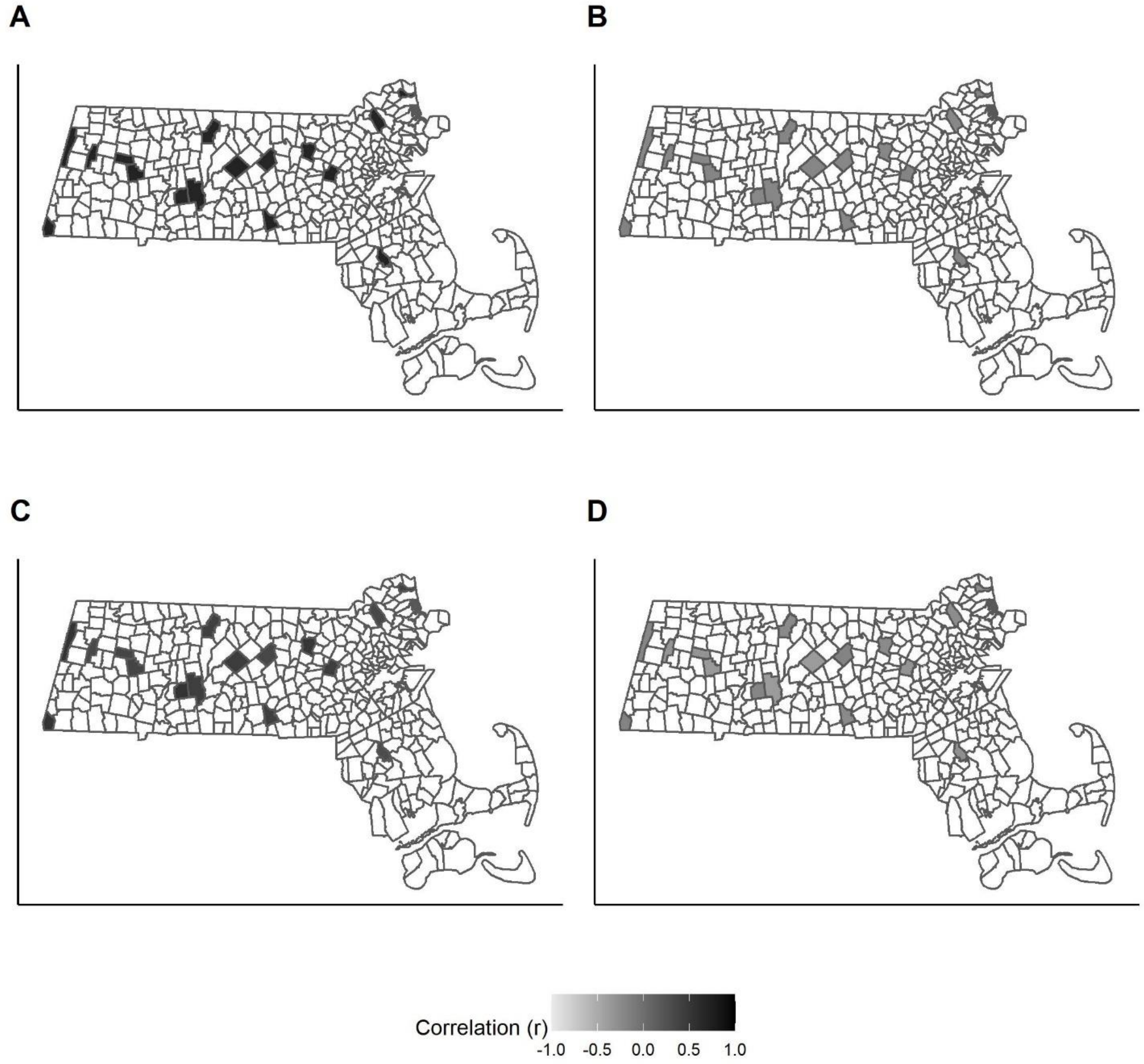

S4 Fig 11. Correlation coefficients between town-level BBS and eBird detection probability indices across Massachusetts for Red-bellied Woodpecker (*Melanerpes carolinus*). (A, B) compare BBS with eBird GLMM methods, and (C, D) compare BBS with eBird RF methods. Left-sided maps (A, C) compare annual estimates and right-sided maps compare inter-annual changes in these estimates.

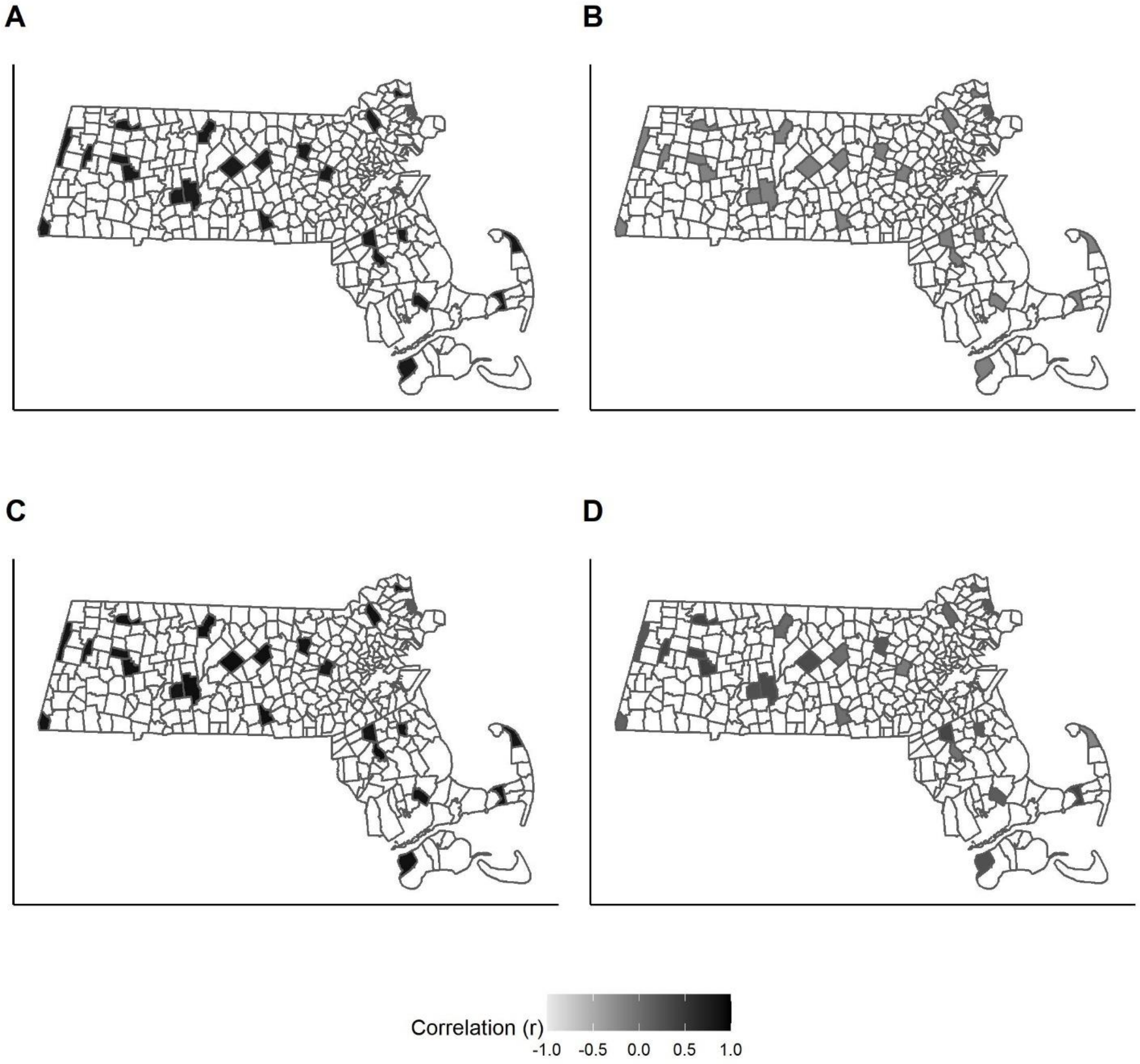

S4 Fig 12. Correlation coefficients between town-level BBS and eBird detection probability indices across Massachusetts for Red-tailed Hawk (*Buteo jamaicensis*). (A, B) compare BBS with eBird GLMM methods, and (C, D) compare BBS with eBird RF methods. Left-sided maps (A, C) compare annual estimates and right-sided maps compare inter-annual changes in these estimates.

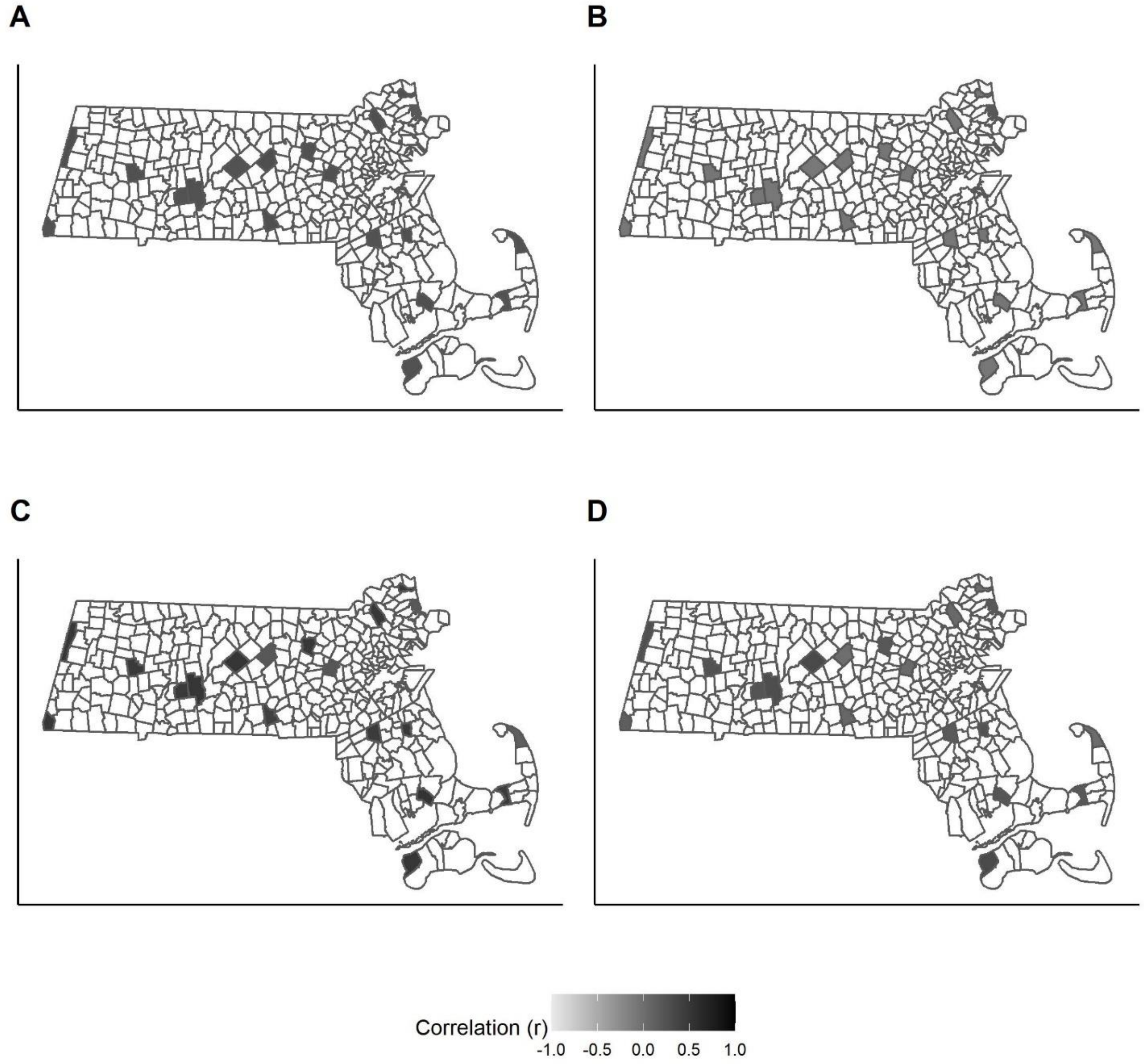

S4 Fig 13. Correlation coefficients between town-level BBS and eBird detection probability indices across Massachusetts for Red-winged Blackbird (*Agelaius phoeniceus*). (A, B) compare BBS with eBird GLMM methods, and (C, D) compare BBS with eBird RF methods. Left-sided maps (A, C) compare annual estimates and right-sided maps compare inter-annual changes in these estimates.

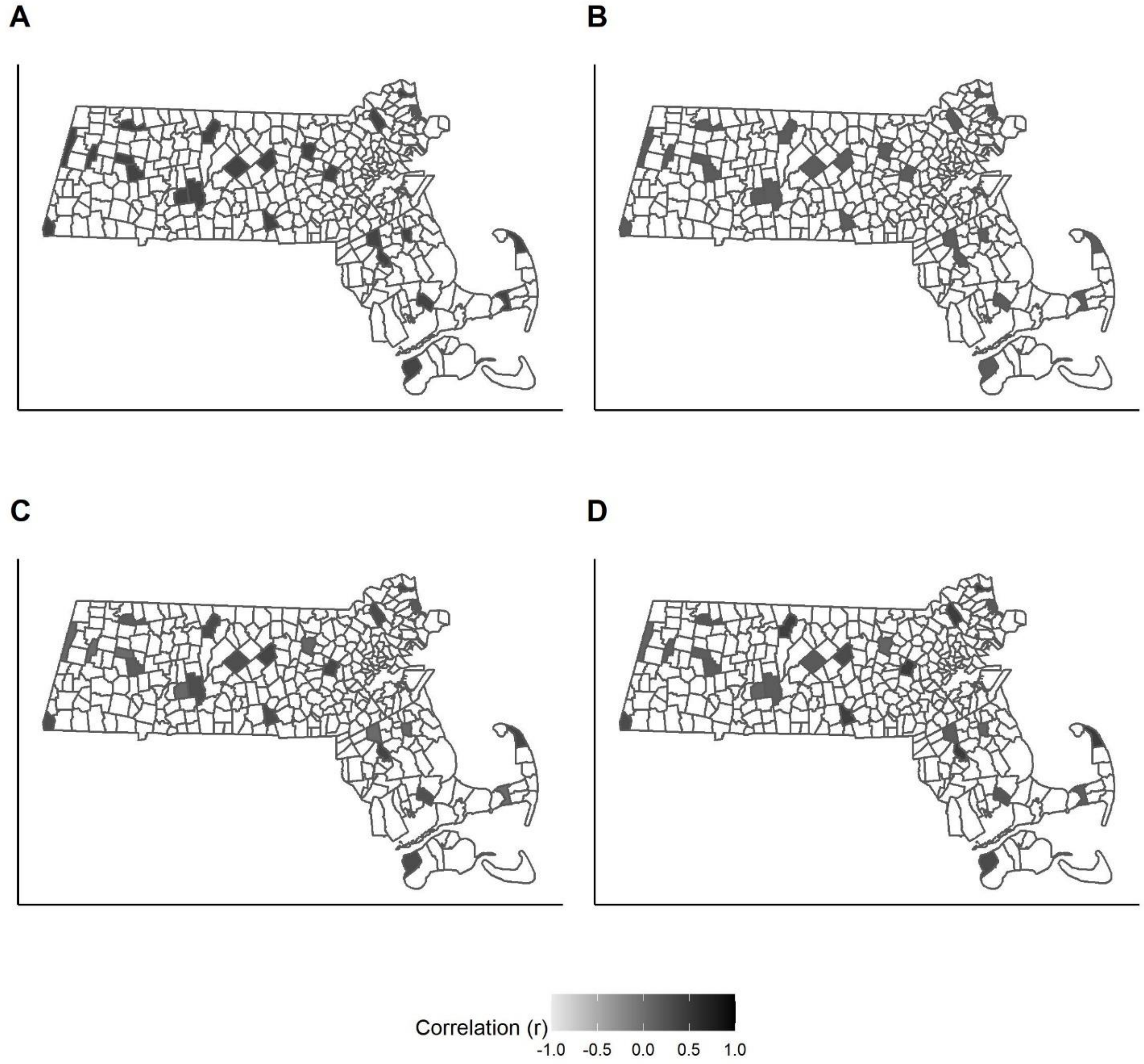

S4 Fig 14. Correlation coefficients between town-level BBS and eBird detection probability indices across Massachusetts for Turkey Vulture (*Cathartes aura*). (A, B) compare BBS with eBird GLMM methods, and (C, D) compare BBS with eBird RF methods. Left-sided maps (A, C) compare annual estimates and right-sided maps compare inter-annual changes in these estimates.

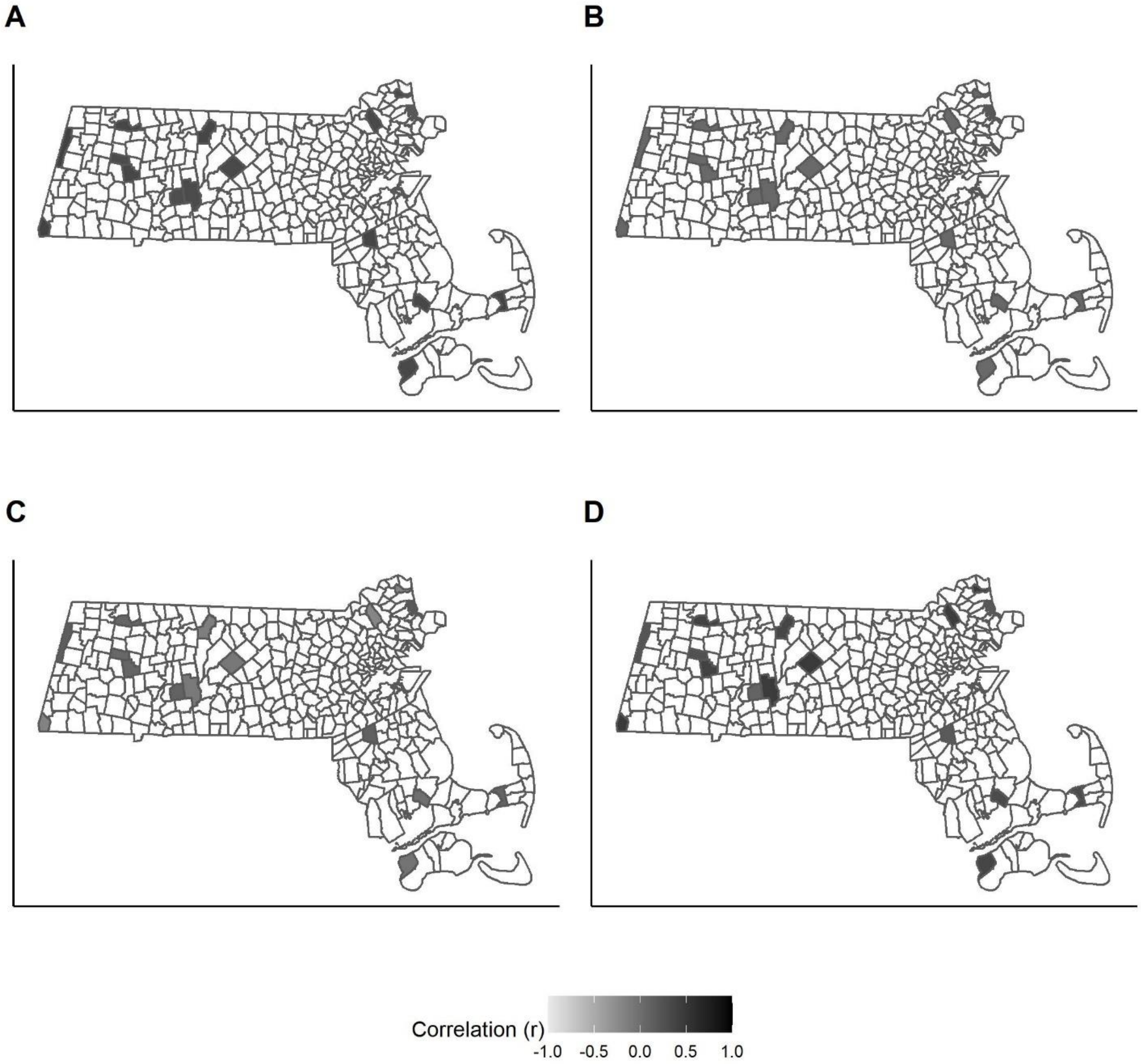

Supplement: S4 Appendix — Massachusetts town maps colored by their Spearman Rank Correlation coefficients of relative abundance estimates between eBird and BBS datasets. (PDF) [file pone.0257226.s004.pdf]
