# Supplementary material for: Comparing the reliability of relative bird abundance indices from standardized surveys and community science data at finer resolutions
Source: PLoS One. 2021 Sep 10;16(9):e0257226. doi: 10.1371/journal.pone.0257226 (PMC8432801; doi:10.1371/journal.pone.0257226)
Supplement: Attachment — Submitted filename: Response to Reviewers.pdf [file pone.0257226.s006.pdf]

Response to Reviewers

Mei-Ling Emily Feng[1*], Judy Che-Castaldo[1]

1 Alexander Center for Applied Population Biology, Lincoln Park Zoo, Chicago, Illinois, United States of America

* mefeng7@gmail.com

Reviewer 1

0.1 Main Comments

Multiple studies have compared standardized bird survey data with community science (eBird) data at coarse spatial scales. This study aims to compare these data sources at a finer scale. The study compares two Breeding Bird Survey indices with two eBird indices during the breeding season in Massachusetts. Correlation was highest between the two BBS indices, moderate between the two eBird indices, and relatively low among data sources, particularly for inter-annual changes in relative abundance.

I appreciate the ideas behind this study. In an era when vast amounts of community science data are becoming available it is critical to make comparisons with standardized data sources. For the most part I think the methods are suitable, although important clarifications are needed in several places. My main concerns are ones of scope, and the generality of the findings.

First, the focal species give cause for concern. They appear to have been selected for another, very different, study investigating birds that can cause electrical outages! This does not seem to be the best criterion for a study testing data reliability. The result is just 14 species from eight families. Five species are woodpeckers, three species are icterids, and three are raptors, showing high taxonomic bias. Two species are also invasive, which is interesting but never mentioned. To fully realize the aims of the study, this limited species list does not seem appropriate. To make reasonable inferences I would want to see many more species of higher taxonomic and ecological diversity. There are no study limitations that preclude the analysis of more species; the eBird data are available on all.

We thank Reviewer 1 for their favorable comments and constructive feedback. We appreciate their suggestions for improving the scope and robustness of our findings.

Their first comment relates to the selection of our study species. The reviewer correctly states that our study species are limited and have taxonomic bias towards select functional groups known to cause electrical outages. We conducted this analysis of eBird data with the primary focus of assessing its reliability at finer spatiotemporal resolutions for usage in an electrical outage case study. Although our sample size is not large, our study species represent a wide range of factors known to influence detectability and data reliability, and we find relatively consistent results across these species. For example, they cover a broad spectrum of prevalence and detectability (as seen in Table 1), functional distinctness based on life histories such as nesting habitat (cavity, tree, shrub, cliff nesting), foraging strategy (ground, bark, soaring, diving), and diet (insectivore, carnivore, scavenger, granivore), variety in conservation status (invasives such as European Starling, and conservation successes such as Osprey), and

occupy a variety of habitats (forest interior, coastal and open water, open habitat, and developed land).

Nevertheless, we recognize that this is a subset of all possible species and we mean to present this as a case study that highlights the need for increased vigilance as future bird population studies consider various modeling applications of eBird data, rather than an assessment of all available eBird data. We clarified the wording of our study scope in the introduction (lines 46-48; note that line numbers refer to revised version without track changes), methods (lines 79-80), and discussion lines (366-370), and provide further details of the taxonomic bias, but also diversity of detectability in our study species on lines 96-103 and 364-365.

Second, the aims were to investigate reliability at finer spatio-temporal resolutions. I think the study does a good job at the temporal aspect. However, simply restricting the study to Mass in my opinion falls short of investigating spatial variation in reliability. I think it would be awesome to test reliability state-by-state, but I understand that might be too large in scope. But two ideas the authors could consider are assessing reliability by habitat, and assessing differences in reliability between towns. I can imagine a map of towns across Mass with each colored by the degree of correlation between indices. It brings to mind the paper by Jarzyna et al (2015, Global Ecol. Biogeogr.) looking at the spatial scaling of temporal changes in New York State.

We thank the reviewer for this suggestion, and we agree that a more explicit comparison at a smaller spatial scale would enhance our study. Following the reviewer's suggestion, we added an analysis of whether local spatial attributes such as habitat cover and eBird survey density would impact the survey accuracy of either dataset and indirectly influence the correspondence of indices between them. We added this as an additional section in our results and discussion looking at finer spatiotemporal resolutions (annual and inter-annual, town-level estimates).

For this, we extracted town-level index predictions for each Massachusetts town (i.e., before averaging them for statewide indices) and calculated Spearman Rank correlations between indices at these finer spatial units. We used BBS detection probability as our BBS relative abundance index, since BBS count and detection yielded similar estimates, and compared it with both eBird indices. We mapped the correlation coefficients between BBS detection probability and each eBird index in each town to visualize the spatial variation in correspondence between these datasets. These maps showed that the degree of correspondence between annual indices and their inter-annual changes varied between towns across the state. The extent of this variance differed between species and the indices being compared. We compiled the maps in an additional supplemental document (S4 Appendix) (lines 496-500).

We tested the relationship between index reliability and habitat attributes using a linear mixed model. We used the town-level correlation coefficients as a response, and the spatial features of each town including the density of eBird checklist locations, and the proportion of deciduous forest, developed, wetland, herbaceous, and shrub habitat as fixed predictor variables. Our habitat cover types were extracted using the National Land Cover Database's (NLCD) 2016 release and aggregating wetland, forest, developed, and herbaceous land cover classifications. Since we only had spatial locations for the start of each BBS route, this limited our number of spatial samples across the state. To increase the sample size for analysis, we combined the town-level correlation coefficients across species and included species as a random effect. We found that habitat is a driver of correlation between eBird and BBS annual estimates and anticipate it is due to changes in survey accuracy between different habitat types [1–6]. We add these additional questions and methods to the introduction (lines 46-57) and methods (lines 267-286) sections and report our findings in the results (lines 335-354,

Figure 4, Table 4) and discussion (lines 443-452).

We address additional clarifications needed in the methods in the minor comments section below.

0.2 Minor Comments

Title: *"The title seems a little vague; it doesn't really give a sense of the paper. Perhaps something like: 'Comparing the reliability of bird abundance indices between standardized surveys and community science data at fine-scale temporal resolutions'."* We changed the title from "Reliability of relative bird abundance indices at fine-scale temporal resolutions" to "Comparing the reliability of relative bird abundance indices from standardized surveys and community science data at finer resolutions."

L31: *The fact that eBird data can capture intra-annual changes in abundance is mentioned a couple of times, but how can one compare intra-annual abundance changes between community science data and standardized survey data? The latter have narrow temporal windows (the breeding season, or Christmas). This is not really an issue the paper addresses.*
The reviewer raises an important issue of finding validation data for finer temporal resolutions (intra-annual changes). Previous studies have addressed this issue by validating estimates with with localized monitoring/survey efforts [10, 11]. These data are typically finer resolution studies at smaller scales and extents. We add note of this validation data issue for future applications of these data on lines 455-459 in the discussion. We broaden our wording when describing the finer resolutions of eBird data in the introduction (lines 24, 30-31) because we do not address these inter-annual trends in our study due to the limitation in available intra-annual validation data.

L58, L64: *Why not 2005-2020? eBird data have increased exponentially, so two years could make a big difference. Moreover, when estimating temporal trends over such short time spans, every year counts!*
At the start of this project (April 2020), eBird data were only available through May of 2019, leaving 2018 as the last breeding season with complete data collection for both eBird and BBS.

L68-69: *How does the reliability of abundance indices vary by habitat?*
We have added an analysis to address this question, please see our response to Main Comments section, comment 2.

L73: *14 is not many species and, given the aims of this paper, the subset of species chosen (those that can cause electrical outages) does not seem relevant or representative.*
please see our response to Main Comments section, comment 1.

L101: *This criterion might not be stringent enough. Even if I'm looking at a target species, I might add a house finch or starling as it flies over. Perhaps ≥ 5 would be a better cut-off. Horns et al. (reference 18) use ≥ 4.*
As suggested, we have adjusted the eBird checklist cut-off to include only checklists with ≥ 5 species reported (line 115). This reduced the data that were available for our subsequent models and analyses. Species prevalence (frequency of detections) in each dataset increased for all species except house sparrow (these changes were made to Table 1). We adjusted all figures, tables, and supplemental materials with these updated results. Our overall findings did not change, but the correlation coefficients and p-values reported in our results section (lines 292-299) were also updated.

L135-136: *Geographically, did BBS survey locations overlap well with eBird locations? Birders may be biased towards certain diversity hotspots, and trends in these hotspots may differ from more general locations.*
There were fewer BBS survey locations (route starting locations) (n=25) than eBird survey locations across the state. BBS surveys were also more evenly spatially distributed compared to eBird which tended to be spatially biased near urban areas and along road ways. To see if towns with eBird "hotspots" affected the degree of correspondence between eBird and BBS indices, we also included the relative density of eBird survey locations within each town as a predictor of the correlation between indices (see our response to Main Comment section, comment 2).

Table1: *It is notable how much higher the eBird detection rates are compared to the BBS detection rates. Why is that?*
This seems to be a common discrepancy found between these two datasets. Walker and Taylor (2017) find similar results in their detection rates for multiple study species as well. We are unsure of the cause for this discrepancy, but sampling frequency and protocol differ greatly between these datasets which could impact detection rates.

L147: *I realize that list length is a wrapper for a lot of different effort variables, but it is worth checking to make sure that additional variation cannot be explained by distance, duration etc. Perhaps concordance would increase with more effort accounted for.*
For each species, we compared models that included additional effort variables (e.g., survey distance, duration, number of observers) against models including only list length. Using the R-package AICcmodavg, we compared models using a model selection table that ranked models by the Second-order Akaike Information Criterion (AICc). We found that the latter models out performed accounting for additional effort variables. We added this clarification to the methods section on lines 163-171.

L152-153: *Is a linear effect of DOY a reasonable assumption? Could there not be a peak in activity during the breeding season? In which case it might be better to include a quadratic term.*
For each species, we compared models that included day of year as a quadratic term, day of year as a linear effect, the untransformed list length and also the log of list length. We compared models using the same model selection table mentioned above. Models that used linear effects of DOY were ranked higher than quadratic effects for all species. List length and log(list length) models varied between species and were selected on a species by species basis. We added this information to the methods section on lines 163-171.

L166-167: *It is unclear to me what the difference is between detection probability and the probability of encountering a species on an eBird checklist? Are they not both simply the proportion of checklists on which a given species was detected/encountered?*
The reviewer is correct. These indices both measure the proportion/probability of surveys with species detections. Our intention for these two indices was to test the reliability across two different modeling methods that try to capture the same estimates. We added clarification of this in the methods section on lines 159-160.

L172-175: *The main difference between the detection probability approach and the encounter rate approach seems to be the models used (GLMM vs random forest) and the variables in those models, rather than the response variables? I am probably missing something but it is unclear.*

We added clarification in our methods section (lines 159-160) and aims (lines 54-56) that the selection of detection probability and encounter rates as our metrics were to compare two different modeling methodologies that try to capture the same response (the likelihood of detecting a species on a standardized survey). We expected these methods to capture the same response and show similar trends, however our estimates showed moderate to no correlation between these indices' inter-annual trends. We conclude that modeling methodology can impact the reliability of abundance indices, and discuss these points in lines 376-400.

L192: *What distribution was fitted to the detection data?*
We used a binomial distribution for the BBS detection data, similar to the eBird detection data. We added this clarification to the manuscript (line 219).

L201-201: *In the first set of comparisons, it does not say what is actually compared: temporal trends, annual estimates, or inter-annual changes? From the results it seems to be the latter two.*
Based on the changes in our methods and aims (mentioned above), we restructured our results section to the following order:

Comparing indices within the same dataset at three temporal resolutions (multi-year trends, annual estimates, inter-annual changes) (lines 288-308).

Comparing indices across different datasets at three temporal resolutions (lines 309-325).

Comparing indices across different datasets at finer spatial (town boundaries) and temporal resolutions (annual estimates and inter-annual changes) (lines 335-354).

L229-230: *Where did this p-value come from? I can't see in the methods what test was used to assess the relationship, only that correlation coefficients were calculated.*
To quantify the reliability between relative abundance indices, we calculated correlation coefficients between each pair of indices and assessed their significance using the p-values extracted from the *cor.test* function in base R. We added this to the methods section on lines 231-232.

Figs. 2-4: *The resolution was very low on these figures (a pdf compilation issue I'm sure) making it difficult to really make out what's what.*
We apologize for difficulties that the figure rendering issues may have caused. We confirmed using PACE (Picture Analysis and Conversion Engine) that our figures comply with the specified requirements by the journal (TIF file format within 2250x2625 pixels at 300 DPI).

L292: *Could the results for starlings (here, incorrectly spelled S. vularis) be thrown off by the fact that they are flocking?*
In our updated results, the BBS indices for *S. vulgaris* and *A.phoeniceus* showed the weakest correspondence between annual estimates and inter-annual changes accordingly (lines 294-299). [7] supports the hypothesis that flocking species (such as *S. vulgaris* and *A.phoeniceus*) tend to have higher observational errors and lower accuracy because counting flocks requires greater observer skill. We add this supporting hypothesis to our discussion on lines 373-376. We also correct the spelling for *S. vulgaris*.

L294-295: *Why might they differ? It seems crucial to interpret these discrepancies.*
We interpret the discrepancies between eBird modeling methods on lines 381-400. We

attribute these differences to the data assumptions in the modeling methods themselves (e.g., accounting for hierarchical patterns using GLMMs) and in the amount of data pre-processing used to remove spatiotemporal and sampling bias.

L209-304: *Could one combine the best aspects of both modeling processes?*
We highly recommend using the data preparation best practices outlined in the encounter rate methodology (Strimas-Mackey et al. 2020) to further balance the data before applying it to any model, including the GLMM approach. We add this as a suggestion for future eBird modeling efforts on lines 398-400.

L305-306: *It is worth noting that eBird data do not do a great job of predicting population trends globally (Neate-Clegg et al. 2020, Biological Conservation).*
We add this citation [9] to our discussion on line 405 to support our claim that scales and resolutions impact the reliability of predicted trends from eBird data.

L348-351: *So why not do that?*
We intended for our analysis to represent a case study using a subset of focal species. Please see our response to Main Comments section, comment 1 for additional details.

L362: *What should we conclude about which eBird index is best?*
Our results do not show that one eBird index is better than the other. Neither index showed strong correspondence with BBS at finer spatial and temporal resolutions and the degree of correspondence also varied across species. We add mention of this on lines 384-388 and also suggest that each modeling method may offer advantages at different resolutions as supported by their use in previous studies (Walker and Taylor 2017, Horns et al. 2018, Strimas-Mackey et al. 2020) [8].

Reviewer 2

0.3 Main Comments

It is an interesting study that compares four indices of relative abundance of birds obtained from two data sets (eBird and BBS) over several years. The manuscript is technically sound, and the data support the conclusions well. I believe that the statistical analysis was carried out properly and rigorously. Los autores hicieron que todos los datos subyacentes a los hallazgos en su manuscrito estuvieran completamente disponibles.

We thank Reviewer 2 for their positive comments and support.

Additional references added based on reviewer comments:

References

1. Rigby EA, Johnson DH. Factors affecting detection probability, effective area surveyed, and species misidentification in grassland bird point counts. The Condor. 2019;121(3). doi:10.1093/condor/duz030.

2. Alldredge MW, Simons TR, Pollock KH. Factors affecting aural detections of songbirds. Ecological Applications. 2007;17(3):948–955. doi:https://doi.org/10.1890/06-0685.
3. Pacifici K, Simons TR, Pollock KH. Effects of vegetation and background noise on the detection process in auditory avian point-count surveys. The Auk. 2008;125(3):600–607. doi:10.1525/auk.2008.07078.
4. Diefenbach DR, Brauning DW, Mattice JA. Variability in grassland bird counts related to observer differences and species detection rates. The Auk. 2003;120(4):1168–1179. doi:10.1093/auk/120.4.1168.
5. Kułaga K, Budka M. Bird species detection by an observer and an autonomous sound recorder in two different environments: Forest and farmland. PLOS ONE. 2019;14(2):e0211970. doi:10.1371/journal.pone.0211970.
6. Lele SR, Moreno M, Bayne E. Dealing with detection error in site occupancy surveys: what can we do with a single survey? Journal of Plant Ecology. 2012;5(1):22–31. doi:10.1093/jpe/rtr042.
7. Ganter B, Madsen J. An examination of methods to estimate population size in wintering geese. Bird Study. 2001;48(1):13.
8. James G, Witten D, Hastie T, Tibshirani R. An introduction to statistical learning with applications in R. vol. 103 of Springer Texts in Statistics. 1st ed. New York, NY: Springer Science+Business Media; 2013. Available from: `https://doi.org/10.1007/978-1-4614-7138-7`.
9. Neate-Clegg MHC, Horns JJ, Alder FR, Kemahlı Aytekin MC, Şekercioğlu CH. Monitoring the world's bird populations with community science data. Biological Conservation;248.
10. Furnas B. Rapid and varied responses of songbirds to climate change in California coniferous forests. Biological Conservation. 2020;241:108347. doi:10.1016/j.biocon.2019.108347.
11. Heim W, Heim R, Beermann I, Burkovskiy O, Gerasimov Y, Ktitorov P, et al. Using geolocator tracking data and ringing archives to validate citizen-science based seasonal predictions of bird distribution in a data-poor region. Global Ecology and Conservation. 2020;24:e01215. doi:10.1016/j.gecco.2020.e01215.
