# Supplementary material for: Comparing the reliability of relative bird abundance indices from standardized surveys and community science data at finer resolutions
Source: PLoS One. 2021 Sep 10;16(9):e0257226. doi: 10.1371/journal.pone.0257226 (PMC8432801; doi:10.1371/journal.pone.0257226)
Supplement: Attachment — Submitted filename: Response to Reviewers.pdf [file pone.0257226.s007.pdf]

August 21, 2021

Dear Dr. Paiva Silva and our Reviewers,

We thank you for your continued consideration of our manuscript for publication with PLOS ONE as well as the favorable comments and helpful suggestions. Our adjustments based on reviewer comments have improved the the consistency of our methods and overall interpretation of our results. Following this round of feedback, we now apply the same data preparation methods for both eBird indices, instead of strictly following the existing protocols for each method, so that only the modeling methods differ between the indices. This includes filtering the eBird data by effort variables, using the same time window of observations (May-July), sub-sampling to reduce spatiotemporal bias and class imbalance (ratio of detections to non-detections), and balancing sample sizes across years. In addition to data preparation, we also adjusted the covariates used in the two eBird models (Generalized Linear Mixed Models and Random Forests) to include the same effort variables (list length, protocol type, list length * protocol type, time of day, and day of year). We go into more details on these changes in the comments below. This standardization allowed us to attribute any differences between the indices to the modeling method rather than inconsistencies in the input data or model covariates. Through this process we also updated our R-script to eliminate the unnecessary step of filling in data for unsurveyed towns, which we realized were generating false non-detections in our model training data.

These changes have altered our model performance and results in subsequent analyses, and we have revised our manuscript accordingly. Specifically, the overall RF model performance decreased, so that while AUC values still indicated substantial agreement between model predictions and the testing data, there was only moderate agreement when looking at maximized Kappa (lines 212-214). Although our estimates changed as a result of these model and data adjustments (several species showing improved consistency between annual estimates), our overall findings remained the same: species with strong multi-year trends showed the strongest agreement between annual estimate across datasets and modeling methods. We reported our new findings on lines 312-369 and updated figures and tables along with the abstract and discussion (lines 386-404, 416-428) to reflect the new results. Specifically, we found that Osprey no longer showed consistent estimates across all indices and the eBird GLMM method was on average more consistent with BBS indices. We interpret this last result as evidence that maintaining consistent modeling approaches can improve the reliability of relative abundance estimates across datasets, at least at coarser temporal resolutions.

Thank you for your time and contributions to our manuscript.

Sincerely,
Mei-Ling Emily Feng

Research Associate
Alexander Center for Applied Population Biology
Lincoln Park Zoo
Chicago, IL 60614
mefeng7@gmail.com

*The following reviewer comments are in *italics* and responses are unitalicized.

Reviewer 1

0.1 Minor Comments

L31-33: *I know what point is being made here, that eBird checklists are generally reduced to presence/absence. But eBird data do contain counts, and if eBird users submitted more checklists with more counts instead of 'X's we'd be able to leverage that for relative abundance. Here I would just clarify that, while eBird checklists can provide count data, they are generally reduced to presence/absence due to gaps in the count data.*

We added this clarification on the limited availability of eBird count data to lines 32 and 33.

L199: *I am curious what the reasoning is about including effort in the random forest models but not the GLMMs? Is it that random forests can take all the variables because it assesses their relative importance while in GLMMs you need to choose a best candidate model? Because the two approaches differ in both modelling framework (GLMM vs RF) and model covariates, its difficult to assess whether discrepancies between the estimates result from the former or the latter.*

We followed the RF methodology described in the eBird Best Practices [1] which includes multiple effort variables, and additionally included list length because it was shown to be representative of multiple effort covariates in previous eBird abundance models [2, 3]. We verified this finding using the RFs ability to assess variable importance. When we assessed the relative importance of each effort variable in the RF, list length was the most important effort variable, consistent across all species.

Because of this and based on the reviewer's comment, we recognize it may be redundant to include the other effort variables as well as list length. We now include only list length and protocol type as effort variables in the RF models (removing observer ID, survey duration, distance, and number of observers). The new model was Detection $\sim$ Year + Day of Year + Time of Survey + Protocol Type + List Length + Town, and we found little change in model performance compared to models using all effort variables (less than a 3% change in MSE, Sensitivity, Specificity, AUC, Kappa values) for all species models. We now use this same set of predictors in both the eBird RF and GLMM models, which should limit the discrepancies between the RF and GLMM methods to be due only to differences in model type rather than covariate selection.

We added all the above mentioned changes to the RF methods section on lines 203-208 and to S1 Appendix which describes the details of our RF method.

L222: *Could list-length analysis be relevant here? BBS participants likely differ in ability, and some may be more likely to detect species than others.*

We do not include list length as an effort variable in BBS models because the BBS conducts structured surveys in order to account for survey effort. We do include observer ID as a random effect to take into account any additional differences between observer skill. We make note of this on lines 224-227.

L239-240: *I am still unsure about distinguishing detection probabilities from encounter rates, although I appreciate the clarification. The authors acknowledge that they "both measure the proportion/probability of surveys with species detections." I therefore think it is misleading to redefine the two terms when they mean the same thing. Its totally reasonable to use different modelling approaches on the same response variable, and they could be referred to as detection probability GLMM vs detection probability random forest (DP-GLMM vs DP-RF). To think of an analogy from species distribution modelling, often different modelling approaches are compared (e.g. GLMM, GAM, RF, Maxent) on the same response variable (presence/absence). In those instances, you hear them say the GLMM vs the RF, not the presence/absence model vs the occurrence model. At the very least, I think the text should clarify that the two terms are functionally identical and*

are only differentiated to aid with communication.

We thank the reviewer for their help clarifying our terminology for readers. We make their suggested change and instead of referring to RF estimates as ”encounter rates” and GLMM estimates as ”detection probabilities” we refer to both by their model frameworks rather than their outputs. All instances of ”encounter rate” and ”detection probability” in the manuscript text, tables, and figures were adjusted to ”detection probability” using either the RF or GLMM method (abbreviated as ”DP-RF” and ”DP-GLMM”).

Reviewer 3

0.2 Main Comments

The authors first use four different models to calculate relative abundance indices from eBird and BBS data. The main portion of the work then involves either (1) the use of these indices in correlation tests to assess the consistency in estimates across models and data types; (2) using these indices in simple linear models to estimate temporal trends (i.e., Table 2); or (3) deriving correlation coefficients for these indices and subsequently using the correlation coefficients in linear mixed models to assess effects of habitat type and eBird survey density on these correlations. However, the relative abundance indices have uncertainty associated with them from the model outputs, and it is probably likely the amount of uncertainty varies across the different modeling approaches, species, and data sets. A proper treatment of uncertainty would then propagate the uncertainty in these indices into further analyses that use these results. By treating the relative abundance indices from the model output as known, the authors ignore this uncertainty. This may not have an influence on the overall value of resulting ”downstream” statistical tests (e.g., the estimated value of the correlation coefficient or temporal trend likely would not change), but it likely has a large influence on the uncertainty (and thus statistical significance) on these values. This is particularly true for the linear mixed model analysis in lines 342-350, as it does not propagate uncertainty from either the relative abundance indices into the correlation coefficients, or the correlation coefficients into the LMM.

In a frequentist perspective, a bootstrapping approach is a common way to propagate the uncertainty from one analyses to the next. A more simple approach could be to take the relative abundance indices and then weight these values by their standard errors in subsequent linear models that use the relative abundance indices as dependent variables. I believe proper uncertainty propagation from one analysis to the next is necessary to adequately assess the statistical significance of the different patterns the authors are reporting. Performing such an approach would greatly improve my confidence in the underlying results, as well as for many quantitative ecologists that would be interested in this work, and so I'll suggest the authors explore these alternative routes to more accurately account for uncertainty in their analyses. At the very least, the authors should include a paragraph in the discussion describing the lack of uncertainty propagation and the implications this has on the resulting inference.

We thank Reviewer 3 for their feedback and agree that it would be important to account for uncertainty propagation in our analyses. To address this, we first calculated standard errors (SEs) for each town-level estimate. For the GLMMs, we used the *bootMer* function from R-package lme4 [4, 5] to calculate standard errors for the town-level estimates using bootstrapping. For the RF models, we directly extracted SEs from the prediction function (*se.fit* = TRUE in *predict.lm*).

We used the reciprocals of these SEs as weights to calculate weighted correlation coefficients for town-level estimates. We used the *cor.wt* function from the R-package *psych* to implement weighted Pearson correlations. This placed greater weight on estimates with smaller standard errors [3]. We then use these weighted correlation coefficients in our analysis examining whether the correspondence between eBird and BBS indices depended on habitat type and eBird survey

density. To incorporate uncertainty into our state-level analyses, we weighted the town-level estimates with the reciprocals of their SEs, and then averaged the weighted estimates across all towns in the state. We added these methods to lines 233-243.

We address additional clarifications needed in the methods in the minor comments section below.

0.3 Minor Comments

L162-163: *Please state the specific algorithm used to fit the GLMMs, which if the default of the glmer function in lme4 was used is Laplace approximation. While this may be clear to R users, this is not as clear to people using other software, and is important given differences among frequentist approximation methods for GLMMs (Bolker et al., 2009).*

We used a modified Laplace approximation method in which the random effects and fixed-effects coefficients were optimized in the penalized iteratively reweighted least squares step rather than in the nonlinear optimizer (parameter nAGQ set to 0 in function glmm) [5], which allowed our models to converge. We added Bates et al. (2021) and Bolker et al. (2009) to our references and clarify our GLMM approximation method on lines 174-177.

L194: *How was the spatial and temporal subsampling done? Was it done following recommendations in Johnston et al (citation 33)? Clarification of the specific filtering approaches used would be helpful, even if it is just a reference to some other paper.*

The spatial and temporal sub-sampling followed recommendations from Johnston et al. and Strimas-Mackey et al. (2020) [7] [1]. We added these references to the appropriate lines (125-127) and refer to S1 Appendix where these specific sub-sampling and filtering approaches are described in more detail.

L398-400: *Why did the authors not do this in their analyses? It seems odd to compare one model that uses the best practices for eBird data filtering and another model that does not, because then, as the authors mention, differences between the approaches could be a result of the differences in filtering processes, rather than more of an assessment of the actual modeling approach. I understand they are comparing commonly used methods for modeling eBird data, but the GLMM approach was from a paper in 2017, and the eBird best practices have been updated numerous times since then, so why not update the model with the best filtering practices and then compare it to the random forest model?*

We recognize that using the latest best practices for data management is essential for making reliable models, so we now apply the data filtering from the eBird best practices [1] to process the dataset going into both the RF and GLMM models. This includes filtering by the effort variables, as well as sampling the data to reduce spatial and temporal bias and class imbalance in the presence and absence observations and to balance the number of surveys across years. We added this to the methods section on lines 125-127 and to the S1 Appendix and removed this as a suggestion in the discussion.

References

1. Strimas-Mackey M, Hochachka WM, Ruiz-Gutierrez V, Robinson OJ, Miller ET, Auer T, et al. Best Practices for Using eBird Data. Version 1.0. Cornell Lab of Ornithology, Ithaca, New York; 2020. Available from: `https://cornelllabofornithology.github.io/ebird-best-practices/`.

2. Horns JJ, Adler FR, Sekercioglu CH. Using opportunistic citizen science data to estimate avian population trends. Biological Conservation. 2018;221:151–159. doi:10.1016/j.biocon.2018.02.027.

3. Walker J, Taylor P. Using eBird data to model population change of migratory bird species. Avian Conservation and Ecology. 2017;12(1). doi:10.5751/ACE-00960-120104.

4. Bates D, Maechler M, Bolker B, Walker S. Fitting linear mixed-effects models using lme4. Journal of Statistical Software. 2015;67(1):1–48. doi:doi:10.18637/jss.v067.i01.

5. Bates D, Maechler M, Bolker B, Walker S. lme4: Linear Mixed-Effects Models using 'Eigen' and S4; 2021 R package version 1.1-27.1. https://cran.r-project.org/web/packages/lme4/lme4.pdf.

6. Bolker B, Brooks M, Clark C, Geange S, Poulsen J, Stevens H, et al. Generalized Linear Mixed Models: A Practical Guide for Ecology and Evolution. Trends in ecology & evolution (Personal edition). 2009;24:127–35. doi:10.1016/j.tree.2008.10.008.

7. Johnston A, Hochachka W, Strimas-Mackey M, Ruiz Gutierrez V, Robinson O, Miller E, et al. Analytical guidelines to increase the value of citizen science data: using eBird data to estimate species occurrence. bioRxiv. 2020;doi:10.1101/574392.
